# CNCPS-Based Nutrient Fractionation, Ruminal Degradation Kinetics, and Intestinal Amino Acid Flow Estimates in Dairy Feedstuffs

**DOI:** 10.3390/ani16152371

**Published:** 2026-08-03

**Authors:** Yansong Ge, Ziyao Wang, Xu Sun, Xueyan Lin, Zhonghua Wang

**Affiliations:** Key Laboratory of Efficient Utilization of Non-Grain Feed Resources (Co-Construction by Ministry and Province), Ministry of Agriculture and Rural Affairs, Shandong Provincial Key Laboratory of Animal Nutrition and Efficient Feeding, College of Animal Science and Technology, Shandong Agricultural University, Tai’an 271017, China; 15163870688@163.com (Y.G.); wziyao0521@163.com (Z.W.);

**Keywords:** CNCPS, ruminal degradation, protein fractions, feed evaluation, metabolizable protein, intestinal amino acid flows, small intestine, dairy cows

## Abstract

To improve feed evaluation for precision dairy nutrition, this study integrated chemical analysis, CNCPS fractionation, in situ ruminal disappearance, exploratory regression, and amino acid-flow calculations for 19 dairy feedstuffs. Conventional nutrients and CNCPS fractions were determined, and total CP and carbohydrate-fraction disappearance were measured in four ruminally cannulated Holstein cows. Protein-fraction Kd values were inferred by nonlinear fitting with measured CNCPS fractions held constant. In the three silages, non-protein nitrogen represented 93.82–96.80% of soluble CP. Energy feeds had greater total carbohydrate and starch-associated CB1 fractions, whereas cottonseed had the greatest unavailable cell-wall fraction. Exploratory equations for Kd1, Kd2, and Kd3 yielded in-sample R^2^ values of 0.7422, 0.7365, and 0.3871, respectively. Two CNCPS-based sets of intestinally absorbable essential amino acid estimates were then calculated using either in situ-fitted or regression-estimated Kd values. The regression-assisted estimates were generally comparable with those based on in situ-fitted Kd values. However, neither set represented direct intestinal measurements, agreement between them did not constitute validation, and Kp was derived from NRC equations under a standardized dietary scenario. Thus, the amino acid estimates are scenario-dependent, and the regression equations should be considered exploratory pending external validation.

## Simple Summary

Nineteen dairy feedstuffs were characterized for CNCPS carbohydrate and protein fractions. Ruminal disappearance was measured in situ, and fraction-specific degradation constants were fitted. Exploratory regressions related feed composition to protein-fraction Kd values, which were then used, together with in situ-fitted Kd values, to generate two sets of CNCPS-based intestinally absorbable amino acid flow estimates. These estimates were model-derived rather than directly measured. Because the regressions were developed from only 19 feedstuffs and lacked external validation, the equations should be regarded as exploratory relationships, not practical prediction tools.

## 1. Introduction

Accurate feed evaluation is essential in precision dairy nutrition because metabolizable protein and intestinally absorbable essential amino acids influence milk protein synthesis and nitrogen-use efficiency [1,2]. Adequate amino acid supply also contributes to reducing nitrogen excretion [3]. Reliable estimates of ruminal protein degradation and intestinal amino acid supply are therefore required to compare feed protein value, match nutrient supply with animal requirements, and avoid unnecessary dietary CP [4,5].

The CNCPS partitions feed carbohydrate and protein into fractions differing in solubility and ruminal degradation [6], but its outputs depend on feed composition, fraction data, and degradation parameters [7]. Estimates may also vary among feed evaluation systems [8], while metabolizable protein supply and dietary composition can affect nutrient utilization and production responses [9,10]. Existing CNCPS libraries and NRC/NASEM tables provide useful reference values, yet integrated data generated from the same commercial feed samples remain limited, particularly for Chinese feeds and processed by-products. Previous studies have generally focused on individual forages or feed ingredients [11,12]. An integrated in situ and CNCPS approach has also been applied to an individual grain source [13]. However, contemporaneous measurements of chemical composition, CNCPS fractions, fraction-specific in situ Kd values, insoluble-protein amino acid composition, and model-derived intestinal amino acid flows across a common multi-feed dataset remain scarce.

Accordingly, this study evaluated 19 dairy feedstuffs using standardized sampling, analytical, and in situ procedures. The descriptive objectives were to characterize CNCPS carbohydrate and protein fractions and determine fraction-specific ruminal degradation kinetics. The modeling objectives were to explore associations between feed characteristics and fitted protein-fraction Kd values and to calculate scenario-dependent intestinally absorbable essential amino acid flows within the CNCPS framework. The study was intended to provide feedstuff-level integration of batch-derived composition data, representative-composite in situ measurements, amino acid composition, and model outputs, rather than to replace existing feed libraries or establish a universally applicable prediction system.

## 2. Materials and Methods

### 2.1. Evaluation of Carbohydrate and Protein Fractions in Common Ruminant Feedstuffs

#### 2.1.1. Experimental Materials and Feed Sampling

Nineteen feedstuffs commonly used in dairy cow diets were collected or purchased between late May and December 2024 from commercial dairy farms, feed suppliers, or feed-processing enterprises in Shandong, Beijing, Tianjin, Shanxi, and Henan, China. Each feedstuff was represented by at least three independent production or commercial batches. Five spatially separated subsamples from each batch were combined into one composite sample; batch composites were retained and analyzed separately.

The feedstuffs were soybean meal, wheat, flaked corn, wheat bran, extruded soybean hulls, molassed soybean hulls, pelleted sugar beet pulp, dried distiller grains with solubles (DDGS), wheat middlings, cottonseed 1, cottonseed 2, whole-plant corn silage 1, whole-plant corn silage 2, corn stover silage, wheat straw, alfalfa, dried brewer grains, dried distiller grains 1, and dried distiller grains 2. Products sharing a general name but bearing different numerical designations originated from independent sources or processing batches and were treated as distinct commercial feed products rather than biological replicates. Specifically, the two whole-plant corn silages originated from separate commercial sources and ensiling units, the two cottonseed samples from different regions and suppliers, and the two dried distiller grains from different manufacturers and batches; the latter were also distinct from DDGS. Detailed information on sample sources, collection periods, feed types, storage conditions, and processing characteristics is provided in Table A1.

Dry samples were sealed in polyethylene bags and stored at room temperature in a cool, dry, and dark environment. Silages were sealed immediately after collection and stored at −20 °C. Original silage subsamples were used to determine as-fed DM, and the remaining material was freeze-dried, ground, and used for chemical analyses.

#### 2.1.2. Determination Parameters

The determined parameters included dry matter (DM), crude protein (CP), ether extract (EE), ash, neutral detergent fiber (NDF), acid detergent fiber (ADF), lignin, starch, non-protein nitrogen (NPN), soluble crude protein (SCP), neutral detergent insoluble protein (NDIP), and acid detergent insoluble protein (ADIP). All conventional nutrient and CNCPS fraction analyses were performed in triplicate for each independent batch composite.

#### 2.1.3. Major Reagents

Unless otherwise stated, all chemicals and reagents were of analytical grade and were purchased from Sinopharm Chemical Reagent Co., Ltd. (Shanghai, China). These included concentrated sulfuric acid, hydrochloric acid, sodium hydroxide, disodium hydrogen phosphate, sodium dihydrogen phosphate, trichloroacetic acid, perchloric acid, sodium dodecyl sulfate, disodium EDTA, sodium tetraborate, cetyltrimethylammonium bromide, ethylene glycol monoethyl ether, acetone, diethyl ether, and ethanol.

#### 2.1.4. Main Equipment

Major equipment included an analytical balance with a readability of 0.0001 g (Mettler Toledo, Greifensee, Switzerland), a muffle furnace (Nabertherm GmbH, Lilienthal, Germany), a plant sample grinder (Retsch GmbH, Haan, Germany), a fiber digestion apparatus (ANKOM Technology, Macedon, NY, USA), a Soxhlet extraction system and an automatic Kjeldahl nitrogen analyzer (VELP Scientifica Srl, Usmate Velate, Italy), and a UV–visible spectrophotometer (Shimadzu Corporation, Kyoto, Japan). Feed samples used for chemical analyses were ground to pass through a 0.45 mm sieve.

#### 2.1.5. Analytical Methods for Nutrients and CNCPS Fractions 

##### Determination of Conventional Nutrients

The DM contents of feed samples and ruminal residues were determined by oven drying to constant weight according to GB/T 6435-2014 [14]. CP was measured by the Kjeldahl method according to GB/T 6432-2018 [15], using a nitrogen-to-protein conversion factor of 6.25. NDF and ADF were determined using neutral- and acid-detergent extraction procedures according to GB/T 20806-2022 [16] and NY/T 1459-2022 [17], respectively. Crude ash was determined by combustion in a muffle furnace according to GB/T 6438-2007 [18], and EE was measured by solvent extraction according to GB/T 6433-2006 [19]. A summary of the analytical procedures and corresponding methodological standards is provided in Table A6. Acid detergent lignin (ADL) content was determined according to the procedure described by van Soest et al. [20].

##### Determination and Calculation of CNCPS Fractions

Total carbohydrate (CHO) content was calculated on a DM basis as follows:

CHO (% DM) = 100 − CP − EE − Ash

The CNCPS carbohydrate fractions were calculated as follows:

CB1 (% CHO) = Starch (% DM)/CHO (% DM) × 100

CC (% CHO) = [2.4 × ADL (% DM)]/CHO (% DM) × 100

CB2 (% CHO) = {[NDF (% DM) − NDIP (% DM) − 2.4 × ADL (% DM)]/CHO (% DM)} × 100

CA (% CHO) = 100 − CB1 − CB2 − CC

In the present calculations, CB1 was calculated from the measured starch concentration, CC represented the unavailable cell-wall carbohydrate fraction, CB2 represented the potentially available cell-wall carbohydrate fraction, and CA was obtained by difference. The combined non-structural carbohydrate fraction was calculated as:

CNSC (% CHO) = CA + CB1

Starch content was determined using the perchloric acid–anthrone colorimetric method. The CNCPS protein fractions were calculated as follows:

PA (% CP) = NPN (% SCP) × SCP (% CP)/100

PB1 (% CP) = SCP (% CP) − PA (% CP)

Before calculating PB2, PB3, and PC, NDIP and ADIP were converted from a DM basis to a CP basis as follows:

NDIP (% CP) = NDIP (% DM)/CP (% DM) × 100

ADIP (% CP) = ADIP (% DM)/CP (% DM) × 100

The remaining protein fractions were then calculated as:

PC (% CP) = ADIP (% CP)

PB3 (% CP) = NDIP (% CP) − ADIP (% CP)

PB2 (% CP) = 100 − PA − PB1 − PB3 − PC

PA represents the non-protein nitrogen fraction, PB1 the rapidly degradable true protein fraction, PB2 the intermediately degradable protein fraction, PB3 the slowly degradable fiber-bound protein fraction, and PC the unavailable acid-detergent insoluble protein fraction. Non-protein nitrogen, protein soluble in borate–phosphate buffer, neutral detergent insoluble protein, and acid detergent insoluble protein were determined according to van Soest et al. [20].

For both conventional nutrient and CNCPS fraction analyses, each independent batch composite was analyzed in triplicate. The three analytical replicates were averaged to obtain one batch-level value. For each feedstuff, results are presented as the mean ± standard deviation of the independent batch-level values, and analytical replicates were not treated as independent observations.

### 2.2. Evaluation of Ruminal Degradation Rate Constants of Feed Carbohydrate and Protein Fractions

#### 2.2.1. Experimental Materials

The same feedstuffs described in Section 2.1.1 were used. For each feedstuff, the independent batch composites used for chemical composition and CNCPS fraction analyses were combined in equal proportions on a DM basis to prepare one representative feedstuff-level composite sample. This representative composite was used for the subsequent in situ ruminal incubation experiment.

#### 2.2.2. Experimental Animals and Management

Four multiparous, mid-lactation Holstein dairy cows fitted with permanent ruminal cannulas were used in this experiment. The cows had similar physiological status, with body weight, days in milk, milk yield, and dry matter intake of 514 ± 74 kg, 147.46 ± 28.36 d, 42.61 ± 6.80 kg/d, and 26.39 ± 4.20 kg/d, respectively. The cows were clinically healthy and were housed and managed under the same feeding conditions throughout the experimental period. The cows were fed a lactation-period total mixed ration twice daily at 08:30 and 15:30 and milked twice daily at 06:30 and 18:30. The forage-to-concentrate ratio of the basal diet was 41.62:58.38 on a DM basis, and the ingredient composition and nutrient levels of the basal diet are presented in Table A2. All cows had free access to water. Ruminal pH was measured during the experimental period using a calibrated portable pH meter (Mettler Toledo, Greifensee, Switzerland). The ruminal pH ranged from 6.2 to 6.8, which was within the normal physiological range for dairy cows. The ruminally cannulated cows showed normal feed intake, rumination, and health status throughout the experiment.

#### 2.2.3. In Situ Nylon Bag Procedure

Ruminal degradation was determined using the in situ nylon bag technique. Representative composites were ground through a 2.0 mm screen to standardize preparation and limit particle loss through the 50 μm bag pores. Concentrate (5 g) or roughage (3 g) samples were weighed into 8 cm × 12 cm nylon bags. Bags containing the same feedstuff were placed in approximately 50 cm nylon mesh carriers, positioned in the ventral rumen, and secured to the cannula.

Nineteen sequential runs were conducted, one feedstuff per run. In each run, all bags were inserted simultaneously into the four cows before morning feeding and removed sequentially at the assigned times. One bag was incubated per cow at each time point; thus, the four cows served as biological replicates for the disappearance measurements, and the bags were measurement units within cows. The final Kd parameters were estimated at the feedstuff level from the mean disappearance series across the four cows, as described in Section 2.2.6. Incubation times were 1, 2, 4, 8, 16, 24, 36, and 48 h for concentrates and 2, 4, 8, 16, 24, 36, 48, and 72 h for roughages; the additional early and late time points captured rapid concentrate and slow roughage disappearance, respectively.

Four zero-hour bags per feedstuff were immediately washed, dried, and weighed without ruminal incubation to quantify procedural washing loss and immediate particle loss. Each run also included four blank bags, one per cow, incubated for the longest assigned time and processed identically to monitor bag-weight changes and background material. Because blank-bag weight changes were negligible, no numerical correction was applied.

Because particle structures differed among feedstuffs, all bags were washed by the same trained operator in five successive 1 min cycles with fresh clean water until the rinse water was clear. Bags were then dried at 65 °C to constant weight for approximately 72 h, weighed, and analyzed as described in Section 2.1.5. Microbial N contamination was corrected using ^15^N-labeled ruminal solid-associated bacteria (SAB). Ruminal microorganisms were initially labeled by continuous intraruminal infusion of ^15^N-labeled ammonium sulfate (99 atom% excess; Cambridge Isotope Laboratories, Inc., Tewksbury, MA, USA) at 3 g/d for 6 d before the first incubation run [21]. Thereafter, the infusion was maintained at the same rate throughout all 19 sequential incubation runs to maintain microbial ^15^N enrichment. In each run, SAB was collected separately from each cow, isolated from solid digesta as described by Legay-Carmier and Bauchart [22], and matched to residues from the same cow. The ^15^N atom-percent excess of residues and SAB was determined using an elemental analyzer (Elementar Analysensysteme GmbH, Langenselbold, Germany) coupled to a DELTA V Advantage isotope-ratio mass spectrometer (Thermo Fisher Scientific, Bremen, Germany). Microbial N was estimated from the ratio of ^15^N atom-percent excess in residual N to that in SAB N and subtracted from total residual N before calculating corrected CP disappearance and degradation kinetics [23].

#### 2.2.4. Experimental Instruments and Reagents

The equipment and reagents used in this experiment were the same as those described in Section 2.1.3 and Section 2.1.4, with the addition of a 2.0 mm screen, ^15^N-labeled ammonium sulfate, an intraruminal infusion apparatus, and the elemental analyzer–isotope-ratio mass spectrometry system described in Section 2.2.3.

#### 2.2.5. Analytical Methods

The determined parameters and analytical methods were the same as those described in Section 2.1.2 and Section 2.1.5.

#### 2.2.6. Calculation Methods

The proportion of microbial N in total residual N was calculated as follows:

P_MN_ = ^15^N′_residue/_^15^N′_SAB_

The amount of microbial N associated with each incubation residue was calculated as follows:

N_microbial_ = N_residue_ × P_MN_

Feed-derived residual N was calculated as follows:

N_feed,residue_ = N_residue_ − N_microbial_

The N disappearance rate corrected for microbial contamination was calculated as follows:

tDR(%) = [(N_initial_ − N_residue_ + N _microbial_)/N_initial_] × 100

where P_MN_ is the proportion of microbial N in total residual N; ^15^N′_residue_ and ^15^N′_SAB_ are the ^15^N atom-percent excess values of residual N and SAB N, respectively; N_initial_ is the initial amount of N placed in the nylon bag; N_residue_ is the total amount of residual N after incubation; N_microbial_ is the estimated amount of microbial N associated with the residue; and N_feed,residue_ is the corrected amount of feed-derived residual N. The corrected N disappearance values were used to calculate corrected CP disappearance and protein degradation kinetics.

The ruminal degradation kinetics were described using the model proposed by Ørskov and McDonald [24]:

*P* = *a* + *b* (1 − *e*^−*ct*^)

where *P* is the degradability at time *t*, *a* is the rapidly soluble fraction, *b* is the slowly degradable fraction, and *c* is the degradation rate constant of fraction *b*.

The zero-hour-bag values were used only to quantify procedural washing loss and were not used to define or replace the chemically determined PA fraction. Blank nylon bags were used to monitor changes in bag mass and background material during incubation, washing, and drying. Because these changes were negligible, no numerical blank-bag correction was applied. Residual N values were corrected for microbial contamination before calculating CP disappearance and fitting the protein degradation curves.

Within the CNCPS framework, crude protein is divided into five fractions: PA, PB1, PB2, PB3, and PC. In this equation, A, B1, B2, and B3 represent the proportions of PA, PB1, PB2, and PB3 in total crude protein, respectively, and are expressed as % CP rather than absolute nutrient amounts. Accordingly, A was fixed exclusively at the experimentally determined PA proportion and was not derived from the zero-hour washing loss. Because fraction PC is considered undegradable in the rumen, the ruminal degradation equation for crude protein was expressed as follows:

P = A + B1 (1 − e^−Kd1t^) + B2 (1 − e^−Kd2t^) + B3 (1 − e^−Kd3t^)

For each feedstuff and incubation time, the corrected total CP disappearance values obtained from the four cows were averaged to generate one feedstuff-level mean disappearance value. The resulting mean disappearance series, Pobs(t), was used as the dependent dataset for a single nonlinear fit for each feedstuff. The experimentally determined proportions of PA, PB1, PB2, and PB3 in total CP were fixed and entered into the model as A, B1, B2, and B3, respectively, whereas PC was excluded because it was considered ruminally undegradable. Kd1, Kd2, and Kd3 were estimated simultaneously by nonlinear least-squares fitting in GraphPad Prism version 5.0 for Windows (GraphPad Software Inc., La Jolla, CA, USA) by minimizing the sum of squared differences between the observed and model-predicted mean CP disappearance values across all incubation times. Thus, the individual protein fractions were not measured separately, and the reported Kd values represent feedstuff-level fitted constants derived from the mean disappearance curves of the four cows rather than means of cow-specific Kd estimates.

For example, soybean meal contained 7.81%, 6.78%, 83.01%, and 0.46% of CP as PA, PB1, PB2, and PB3, respectively. Its fitted equation was therefore:

Ppred(t) = 7.81 + 6.78 (1 − e^−Kd1t^) + 83.01 (1 − e^−Kd2t^) + 0.46(1 − e^−Kd3t^).

The three Kd values were adjusted jointly until the predicted total CP disappearance curve provided the best least-squares fit to the corrected CP disappearance values observed over the complete incubation period. The PC fraction, which accounted for 1.94% of CP in soybean meal, was not included because it was assumed to be undegradable in the rumen.

For carbohydrates, the CNCPS classifies feed carbohydrates into four fractions: CA, CB1, CB2, and CC. Fraction CC is considered unavailable and was therefore not assigned a ruminal degradation rate constant. For each feedstuff and incubation time, the concentrations of CA, CB1, and CB2 in the original feed and incubation residue were calculated using the CNCPS fractionation equations described in Section Determination and Calculation of CNCPS Fractions and were expressed on a DM basis.

The initial amount of each carbohydrate fraction placed in the nylon bag was calculated as follows:

M_initial,i_ = W_initial_ × (C_initial,i/_100)

The amount of the corresponding fraction remaining after incubation time t was calculated as follows:

M_residual,i,t_ = W_residual,t_ × (C_residual,i,t_/100)

The disappearance of each carbohydrate fraction at incubation time t was then calculated as follows:

D_i,t_ (%) = [(M_initial,i_ − M_residual,i,t_)/M_initial,i_] × 100

where M_initial,i_ is the initial amount of carbohydrate fraction i placed in the nylon bag; M_residual,i,t_ is the amount of fraction i remaining after incubation time t; W_initial_ is the initial DM weight of feed placed in the nylon bag; W_residual,t_ is the residual DM weight after incubation, washing, and drying; and C_initial,i_ and C_residual,i,t_ are the concentrations of fraction i in the original feed and incubation residue, respectively, expressed as % DM. Thus, disappearance was calculated from the initial and residual amounts of each fraction rather than from changes in concentration alone.

Analytical variation in residual DM weight or chemical composition could theoretically result in negative disappearance values or values greater than 100%, particularly when the initial amount of a fraction was small. Therefore, all calculated disappearance values were checked against the original bag weights, residual weights, analytical results, and calculation records before nonlinear curve fitting. Suspect values were rechecked using the original laboratory records, and no values were automatically truncated, replaced, or imputed. For each feedstuff and incubation time, the verified carbohydrate-fraction disappearance values obtained from the four cows were averaged. The resulting feedstuff-level mean disappearance series were fitted by nonlinear least-squares regression to estimate Kd4, Kd5, and Kd6 for CA, CB1, and CB2, respectively. Accordingly, the reported carbohydrate-fraction Kd values are feedstuff-level fitted constants rather than means of cow-specific estimates.

### 2.3. Estimation of Intestinally Absorbable Amino Acid Flows Based on the CNCPS Model

#### 2.3.1. Feed Samples for Amino Acid Analysis

The same feedstuffs described in Section 2.1.1 were used.

#### 2.3.2. Amino Acid Analysis Instruments

Amino acid analysis was performed using an L-8800 automatic amino acid analyzer (Hitachi High-Tech Corporation, Tokyo, Japan). The analytical balance and sample-preparation equipment were the same as those described in Section 2.1.4.

#### 2.3.3. Calculation of Intestinally Absorbable Amino Acid Flows

##### Calculation Method of Rumen Passage Rate

The passage-rate equations used in this study were derived from NRC (2001) [25]. The corrected positive sign for the dietary concentrate term in the dry-forage equation was used, and the equations were considered suitable empirical tools for lactating dairy cows based on the evaluation by Seo et al. [26]. Although NASEM (2021) represents the current reference for dairy cattle nutrient requirements [27], the NRC equations were retained in the present study because they provide separate passage-rate estimates for wet forages, dry forages, and concentrates. The following equations were used to calculate Kp:

For wet roughages, such as silage and fresh forage:

Kp = 3.054 + 0.614x_1_

For estimating dry roughages:

Kp = 3.362 + 0.479x_1_ + 0.007x_2_ − 0.017x_3_

For estimating concentrates:

Kp = 2.904 + 1.375x_1_ − 0.020x_2_

where Kp is the fractional ruminal passage rate (%/h); x_1_ is DMI expressed as a percentage of BW; x_2_ is the proportion of concentrate in dietary DM (%); and x_3_ is the NDF concentration of the individual dry forage (% DM).

For the standardized model calculations, DMI was set at 4% of BW and the dietary concentrate proportion was set at 50% of DM. These rounded values were selected to define a simplified reference scenario for within-study comparisons among feedstuffs and were not derived from the measured intake or diet composition of the donor cows. The donor cows had a mean DMI of 26.39 kg/d and a mean BW of 514 kg, corresponding to approximately 5.13% of BW, and their basal diet contained 58.38% concentrate on a DM basis. Thus, the difference between the observed animal values and the standardized model inputs was intentional. The standardized inputs were retained to evaluate all feedstuffs under identical calculation conditions rather than to estimate animal-specific passage rates. No common dietary NDF value was imposed; for the dry-forage equation, x3 was the measured NDF concentration of each individual dry forage.

Whole-plant corn silage 1, whole-plant corn silage 2, and corn stover silage were classified as wet forages, for which the calculated Kp was 5.5%/h. Wheat straw and alfalfa were classified as dry forages. Because the NRC dry-forage equation incorporates the NDF concentration of the individual feedstuff, the calculated Kp values were 4.3%/h for wheat straw and 4.9%/h for alfalfa. The remaining concentrate-type feedstuffs and by-products were assigned a calculated Kp of 7.4%/h.

These equations were considered appropriate for the present standardized comparisons because the donor animals were multiparous, mid-lactation Holstein cows, which fall within the lactating dairy-cow population for which the equations were developed and evaluated, and because the tested feedstuffs could be classified into the corresponding wet-forage, dry-forage, or concentrate categories. However, the equations do not explicitly account for feed particle characteristics, processing effects, liquid flow, or temporal variation in intake. Moreover, the Kp values were not measured in the donor cows, and the standardized DMI and dietary concentrate inputs were not intended to reproduce their individual passage rates. Therefore, the calculated Kp values were used as standardized scenario inputs rather than as animal- or feed-specific measurements, and the resulting intestinally absorbable amino acid flow estimates should not be generalized to other animals or diets without recalculation.

##### Amino Acid Composition of Ruminal Microbial Protein

The amino acid composition of ruminal microbial cell-wall and non-cell-wall protein used in the CNCPS-based calculations is presented in Table A7. The means and standard deviations for ruminal microbial protein were derived from 441 microbial samples obtained from 61 diets in 35 experiments [28]. Because tryptophan content was not reported separately for the microbial cell-wall fraction, it was assumed to be identical to that of the non-cell-wall fraction [29].

##### Calculation Pathways for Intestinally Absorbable Essential Amino Acid Flows

All intestinally absorbable essential amino acid flows reported in this study were model-derived estimates and were not measured directly in intestinal digesta. Two calculation pathways were used. In the first pathway, measured chemical composition and insoluble-protein amino acid composition, experimentally determined CNCPS protein fractions, NRC-derived ruminal passage rates (Kp) calculated under the standardized dietary scenario, and Kd1, Kd2, and Kd3 values fitted from the in situ ruminal disappearance data were entered into the CNCPS-based calculation framework. These outputs are hereafter referred to as CNCPS-based estimates using in situ-fitted Kd values.

In the second pathway, the same measured inputs, CNCPS fractions, Kp assumptions, and ruminal nitrogen-balance scenarios were retained, but the in situ-fitted Kd1, Kd2, and Kd3 values were replaced by values estimated from the exploratory regression equations reported in Section 3.2.3. These outputs are hereafter referred to as regression-assisted CNCPS estimates using regression-estimated Kd values. The regression equations estimated the protein-fraction degradation rate constants only and did not directly predict intestinal amino acid flows.

The intestinally absorbable flow of each essential amino acid derived from rumen-undegraded feed protein was then calculated using the following equation in both calculation pathways:

REFAAij = AAINSPij × 0.01 × CPj × 10 × [(PB1j/100) × Kpj/(Kd1j + Kpj) + (PB2j/100) × Kpj/(Kd2j + Kpj) + (PB3j/100) × Kpj/(Kd3j + Kpj)]

where REFAAij is the intestinally absorbable flow of amino acid i derived from rumen-undegraded protein of feedstuff j (g/kg DM); AAINSPij is the measured content of amino acid i in the insoluble protein of feedstuff j (g/100 g insoluble protein); CPj is the measured crude protein content of feedstuff j (% DM); PB1j, PB2j, and PB3j are the experimentally determined proportions of the corresponding CNCPS protein fractions (% CP); Kpj is the NRC-derived ruminal passage rate assigned to or calculated for feedstuff j under the standardized dietary scenario (%/h); and Kd1j, Kd2j, and Kd3j are either the in situ-fitted or regression-estimated degradation rate constants used in the corresponding calculation pathway (%/h). The factor 10 converts CP from % DM to g/kg DM. Because fraction-specific amino acid compositions were not measured, the measured insoluble-protein amino acid profile of each feedstuff was applied to the rumen-undegraded portions of PB1, PB2, and PB3 as a proxy for the amino acid composition of rumen-undegraded feed protein [12].

The microbial-protein contribution was calculated within the CNCPS framework under positive ruminal nitrogen balance and ruminal nitrogen deficiency, respectively, and was combined with the rumen-undegraded feed-protein contribution to obtain total intestinally absorbable essential amino acid flow. These two nitrogen-balance conditions were predefined CNCPS calculation scenarios applied to all feedstuffs; ruminal nitrogen balance was not measured in the donor cows, and no experimental nitrogen-deficiency treatment was imposed.

### 2.4. Statistical Analysis

Data are presented as means ± SD unless otherwise stated. For chemical composition and CNCPS fractions, the independent feed batch was the experimental unit, and triplicate laboratory determinations were analytical replicates. For in situ disappearance measurements at each incubation time, cow was the biological replicate. However, the nonlinear degradation parameters were estimated from the mean disappearance series across the four cows for each feedstuff. Therefore, each feedstuff contributed one fitted value for each Kd parameter to the across-feedstuff regression analyses, and between-cow variability in the fitted Kd values was not quantified. Each feedstuff was evaluated in one sequential run using the same four cows; because run was not replicated, run-to-run variation could not be separated from feedstuff effects, and feedstuff comparisons were descriptive unless an inferential test and *p*-value were reported. Regression analyses used one feedstuff-level mean per variable (*n* = 19).

Before analysis and nonlinear fitting, records were checked for completeness, unit consistency, calculation accuracy, duplicate or missing entries, and biologically impossible values, including negative or >100% disappearance and CNCPS-inconsistent fractions. Suspect values were verified against laboratory records. No imputation was used; feedstuffs missing variables required for an analysis were excluded from that analysis. Feed names and units were standardized, and variables were expressed on the appropriate % DM, % CP, or % CHO basis.

GraphPad Prism version 5.0 for Windows (GraphPad Software Inc., La Jolla, CA, USA) was used for nonlinear least-squares fitting of degradation curves. SAS version 9.4 (SAS Institute Inc., Cary, NC, USA) was used for multiple linear regression, principal component analysis, hierarchical cluster analysis, and the associated statistical diagnostics. Exploratory regressions examined (1) relationships of conventional nutrients with CNCPS carbohydrate and protein fractions and (2) relationships of conventional nutrients or CNCPS fractions with fitted Kd1, Kd2, and Kd3. Candidate predictors were DM, CP, NDF, ADF, EE, ash, ADL, starch, NPN, SCP, NDIP, ADIP, and relevant CNCPS fractions. NDIP and ADIP values in Table A3 were converted from % DM to % CP before protein-related regressions.

Predictors were screened manually for statistical association, mathematical independence, collinearity, biological plausibility, and coefficient stability across alternative models and leave-one-observation-out analyses. Biological plausibility required a reasonable relationship with protein solubility, detergent-insoluble protein, cell-wall association, readily fermentable carbohydrate availability, or ruminal accessibility. Variables were rejected when their contribution reflected only improved in-sample fit, mathematical redundancy, an unstable or biologically implausible coefficient direction, or dependence on a single feedstuff or broad feed-category contrast. Final models were selected using overall and predictor significance, in-sample fit, parsimony, biological interpretability, and coefficient stability. No automatic stepwise, forward, or backward selection was used, and no final model contained more than three predictors.

For multiple-predictor equations, VIF < 5 was required. Residual normality was evaluated using the Shapiro–Wilk test and normal Q–Q plots; *p* > 0.05 indicated no significant departure from normality. Residual-versus-fitted plots were used to assess linearity and homoscedasticity. Influential observations were screened using Cook’s distance > 4/*n*, leverage > 2(*p* + 1)/*n*, and |studentized residual| > 2, where *p* was the number of predictors. Flagged observations were examined individually and by leave-one-observation-out sensitivity analysis but were not automatically excluded.

Broad feed category was not included as a predictor because category sizes were small and unbalanced. Residual and influence diagnostics were nevertheless examined by category, and concentrate-type and roughage residuals were compared using Welch’s *t*-test. Model fit was summarized by R^2^, RMSE, MAE, overall model *p*-value, and *n*. Statistical significance was set at *p* < 0.05 and high significance at *p* < 0.01. All regression statistics describe in-sample behavior only; because *n* = 19 and no independent validation dataset was available, the equations were treated as exploratory and not as practical prediction tools.

PCA and hierarchical cluster analysis were conducted using the data in Table 1, Table 2 and Table 3 for CP, NDF, ADF, EE, ash, starch, CA, CB1, CB2, CC, PA, PB1, PB2, PB3, and PC. Variables were standardized using z-scores, and clustering used Euclidean distance and Ward’s method. Because CNCPS carbohydrate and protein fractions sum to 100% within their respective pools, PCA loadings were interpreted as descriptive relative-composition patterns rather than independent fraction effects. These analyses characterized compositional heterogeneity and category-related clustering but were not used for predictor selection or regression construction. Given the small and unbalanced category sizes, separate category-specific models were not fitted.

## 3. Results

### 3.1. Evaluation of Carbohydrate and Protein Fractions of Common Feeds for Ruminants

#### 3.1.1. Proximate Composition Analysis of Common Feeds for Dairy Cows

Table 1 shows that soybean meal had the highest CP content (49.45%), followed by DDGS and dried distiller grains 1 (>30%), whereas alfalfa had the highest CP content among roughages (16.32%). Flaked corn and wheat had the highest starch contents, while wheat straw and extruded soybean hulls had relatively high NDF and ADF contents.

Nitrogen-fraction data are presented in Table A3. Across all feedstuffs, SCP ranged from 3.90 to 81.16% of CP. In the three silages, NPN represented 93.82–96.80% of SCP; among concentrates, SCP represented 10.74–39.89% of CP and NPN represented 26.23–99.78% of SCP. Alfalfa had relatively low ADIP among roughages.

#### 3.1.2. Carbohydrate Content and Proportions of Carbohydrate Fractions in Feeds

Table 2 shows that flaked corn had the highest CHO content (86.13% DM) and CB1 proportion (74.65% CHO) among the energy feeds. Dried brewer grains and extruded soybean hulls had relatively low CB1 proportions. Cottonseed had the highest CC proportions (56.49–63.41% CHO), whereas soybean meal had the lowest (1.03%). Silages and wheat straw generally contained approximately 80% CHO, and wheat straw had a low CB1 proportion.

#### 3.1.3. Proportions of Crude Protein Fractions in Feeds

Protein fractions differed markedly among feedstuffs (Table 3). PA predominated in dried brewer grains and the two whole-plant corn silages and was also high in wheat straw. PB2 dominated most concentrate-type feedstuffs, particularly soybean meal, whereas PC was generally low in concentrates and highest in wheat straw.

#### 3.1.4. Exploratory Regression Relationships Between Conventional Nutrients and CNCPS Fractions

Exploratory regressions were fitted as described in Section 2.4. Models dominated by DM were not retained because DM primarily reflected moisture status and lacked a direct biological relationship with protein solubility or CNCPS fractions. Table A8 therefore presents the biologically interpretable equations with comparatively stronger in-sample fit.

PC and the carbohydrate fractions CA, CB1, CB2, and CC showed the strongest empirical relationships with conventional feed components. CNSC was excluded because of its mathematical dependence on other carbohydrate variables, and soluble-protein models were excluded because of their limited explanatory power; the excluded equations are reported in Table S1. All retained relationships were derived from the 19 tested feedstuffs and were not externally validated.

#### 3.1.5. Exploratory Multivariate Analysis of Feed Nutritional Characteristics

**Figure A1:**
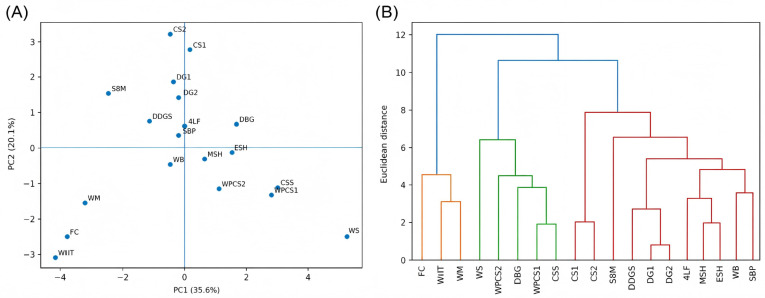
Principal component analysis and hierarchical clustering of the nutritional characteristics of 19 tested feed samples. (**A**) Principal component analysis (PCA) based on standardized conventional nutrient composition, carbohydrate fractions, and protein fractions of the tested feed samples. The first two principal components explained 35.6% and 20.1% of the total variance, respectively. (**B**) Hierarchical clustering analysis based on Euclidean distance and Ward’s method, showing the similarity relationships among feed samples according to their nutritional characteristics. In panel B, the orange, green, and red branches indicate the three clusters formed at lower linkage distances, whereas the blue branches indicate higher-level linkages among these clusters. The branch colors are used only to distinguish the dendrogram clusters and do not represent predefined feed categories. SBM, soybean meal; WHT, wheat; DDGS, dried distiller grains with solubles; MSH, molassed soybean hulls; ESH, extruded soybean hulls; WM, wheat middlings; WB, wheat bran; FC, flaked corn; CS1 and CS2, cottonseed 1 and cottonseed 2; SBP, pelleted sugar beet pulp; WPCS1 and WPCS2, whole-plant corn silage 1 and whole-plant corn silage 2; CSS, corn stover silage; WS, wheat straw; ALF, alfalfa; DBG, dried brewer grains; DG1 and DG2, dried distiller grains 1 and dried distiller grains 2.

The first two principal components explained 55.7% of the total variance, with PC1 and PC2 accounting for 35.6% and 20.1%, respectively (Figure A1A). PC1 contrasted feeds rich in starch and CB1 with those rich in NDF and ADF, whereas PC2 was associated mainly with EE, CP, CC, and PB2. Wheat and flaked corn aligned with starch and CB1; wheat straw, the silages, and soybean hull products aligned with fiber; the two cottonseeds occupied similar positions; and soybean meal was separated by its high CP and PB2 proportions.

Hierarchical clustering grouped wheat, flaked corn, and wheat middlings; cottonseed 1 and cottonseed 2; and dried distiller grains 1 and 2 (Figure A1B). These exploratory groupings indicate compositional similarity rather than nutritional interchangeability.

### 3.2. Evaluation of Rumen Degradation Rates of Carbohydrate and Protein Fractions in Feeds

#### 3.2.1. Study on Rumen Degradation Rate Constants of Protein Fractions in Common Dairy Feeds

Table 4 shows that Kd1 ranged from 37.05 to 396.20%/h, with cottonseed 1 and wheat bran showing the lowest and highest values, respectively. Because Kd1, Kd2, and Kd3 were estimated simultaneously from total corrected CP disappearance while the protein-fraction proportions were held constant, Kd1 values above 100%/h are fitted first-order rate constants rather than percentages degraded within 1 h. Thus, 396.20%/h corresponds to 3.962 h^−1^ and approximately 98.1% modeled disappearance after 1 h. Such values indicate extremely rapid apparent PB1 disappearance but have limited biological precision.

Kd2 ranged from 2.29 to 18.73%/h and largely overlapped the CNCPS PB2 reference range of 3–16%/h. In contrast, Kd3 ranged from 1.35 to 8.16%/h, exceeding the reported PB3 reference range of 0.06–0.55%/h [7].

#### 3.2.2. Study on Rumen Degradation Rate Constants of Carbohydrate Fractions in Common Dairy Cow Feeds

Table 5 shows that Kd4, Kd5, and Kd6 ranged from 3.160 to 214.6%/h, 1.004 to 47.18%/h, and 0.2865 to 4.513%/h, respectively. Flaked corn had the highest Kd4, and Kd5 among the energy feeds ranked as follows: wheat middlings > wheat > wheat bran > flaked corn. All Kd6 values were below 5%/h.

Four curves had R^2^ values below 0.50: the Kd4 curves of whole-plant corn silages 1 and 2 and the Kd6 curves of wheat and cottonseed 2. These estimates are flagged in Table 5 and should not be used for quantitative comparisons among feedstuffs.

#### 3.2.3. Exploratory Regression Relationships Between Feed Characteristics and Protein-Fraction Degradation Rate Constants

Exploratory equations relating feed characteristics to the degradation rate constants of PB1, PB2, and PB3 were fitted as described in Section 2.4:

Kd1 = 262.177 − 7.214 NDIP + 6.570 PB1 − 1.887 CC

(R^2^ = 0.742, RMSE = 42.95, MAE = 33.19, *p* < 0.01, *n* = 19)

Kd2 = 4.721 − 0.188 ADF + 0.850 Ash + 0.226 CA

(R^2^ = 0.737, RMSE = 2.25, MAE = 1.87, *p* < 0.01, *n* = 19)

Kd3 = 2.964 + 0.080 Starch

(R^2^ = 0.387, RMSE = 1.82, MAE = 1.60, *p* < 0.01, *n* = 19)

NDIP in the Kd1 equation was expressed as % CP after conversion from the DM-basis values in Table A3. Kd1 and Kd2 showed stronger in-sample fit than Kd3, whose R^2^ of 0.387 indicated a weak, sample-specific association.

Maximum VIF values were 1.260, 1.385, and 1.000 for Kd1, Kd2, and Kd3, respectively. Residuals did not significantly depart from normality for Kd1 (W = 0.954, *p* = 0.456) or Kd2 (W = 0.957, *p* = 0.515), but did so for Kd3 (W = 0.862, *p* = 0.011). Leave-one-observation-out analyses did not reverse coefficient directions; Kd2 was comparatively stable, whereas the significance of CC in Kd1 and the explanatory performance of Kd3 were more sensitive to individual observations. Residuals did not differ significantly between concentrate-type and roughage feedstuffs (Table A4 and Table A5).

Because no internal or external predictive validation was performed, the equations remain sample-specific empirical associations and should not be applied to new feedstuffs before independent validation.

### 3.3. CNCPS-Based Estimation of Intestinally Absorbable Amino Acid Flows

#### 3.3.1. Amino Acid Composition of Insoluble Crude Protein in Feeds

Table 6 summarizes Lys, Met, and total EAA contents of insoluble protein; complete amino acid profiles are provided in Table S2. Because fraction-specific amino acid profiles were unavailable, each feedstuff’s measured insoluble-protein profile was applied to the rumen-undegraded portions of PB1, PB2, and PB3 as a proxy, without assuming that these fractions had identical amino acid compositions [12].

Soybean meal had the highest Lys and total EAA contents (2.911 and 24.33 g/100 g insoluble protein, respectively), whereas DDGS had the highest Met content (0.366 g/100 g). Wheat straw and molassed soybean hulls had the lowest Lys and Met contents, respectively, and the two whole-plant corn silages and wheat straw had relatively low total EAA contents.

#### 3.3.2. Comparison of Intestinally Absorbable Essential Amino Acid Flow Estimates Using In Situ-Fitted and Regression-Estimated Kd Values

All values in Table 7, Table 8, Table 9 and Table 10 are CNCPS-based estimates rather than direct intestinal measurements. Table 7 and Table 9 use in situ-fitted Kd values, whereas Table 8 and Table 10 use regression-estimated Kd values; all other measured inputs, Kp assumptions, and nitrogen-balance scenarios were identical. Soybean meal had the greatest estimated total intestinally absorbable EAA flow among concentrates, followed by DDGS, whereas alfalfa had the greatest flow among roughages. Under both Kd pathways, the modeled nitrogen-deficiency scenario yielded lower total flows than the positive-nitrogen-balance scenario. Although the regression-assisted estimates were generally comparable with those based on in situ-fitted Kd values, this within-framework agreement does not constitute validation of the regression equations or amino acid flow estimates.

Under positive ruminal nitrogen balance:

**Table 7:** Estimated intestinally absorbable essential amino acid flows using in situ-fitted Kd values under positive ruminal nitrogen balance (g/kg DM).

**Item**	**Soybean** **meal**	**Wheat**	**DDGS**	**Molassed** **Soybean hulls**	**Extruded** **Soybean hulls**	**Wheat** **middlings**	**Alfalfa**
Met	3.598	1.003	1.149	0.770	0.351	1.055	1.039
Lys	13.52	2.969	2.990	2.346	0.966	3.080	3.180
His	4.901	1.023	1.611	0.790	0.320	1.119	1.051
Phe	11.19	2.365	6.180	1.971	1.063	2.561	2.300
Trp	3.116	0.578	0.708	0.524	0.506	0.678	0.648
Thr	9.017	1.986	2.570	1.539	0.583	2.128	2.175
Leu	13.64	2.696	5.807	2.126	0.821	2.999	2.983
Ile	9.846	2.075	2.715	1.616	0.621	2.241	2.293
Val	12.25	2.767	5.605	2.350	1.218	3.102	2.811
Arg	12.18	2.407	3.030	1.910	0.719	2.802	2.674
TEAAA	93.26	19.87	32.37	15.94	7.168	21.77	21.15
**Item**	**Wheat** **bran**	**Flaked** **corn**	**Cottonseed 1**	**Cottonseed 2**	**Pelleted sugar** **beet pulp**	**Whole-plant** **corn silage 1**	
Met	1.190	0.482	0.878	0.674	0.619	0.133	
Lys	3.497	1.279	2.275	1.988	1.821	0.366	
His	1.264	0.463	1.046	0.849	0.623	0.122	
Phe	2.695	1.215	3.230	3.541	1.552	0.292	
Trp	0.722	0.283	0.582	0.496	0.348	0.079	
Thr	2.382	0.902	1.586	1.394	1.212	0.251	
Leu	3.351	1.366	2.327	2.079	1.619	0.343	
Ile	2.536	0.940	1.629	1.455	1.244	0.265	
Val	3.283	1.425	4.284	3.327	1.965	0.341	
Arg	3.172	1.108	3.214	2.595	1.423	0.310	
TEAA	24.09	9.466	21.05	18.40	12.43	2.500	
**Item**	**Whole-plant** **corn silage 2**	**Corn** **stover silage**	**Wheat** **straw**	**Dried** **brewer grains**	**Dried distiller** **grains 1**	**Dried distiller** **grains 2**	
Met	0.178	0.194	0.099	0.404	1.056	0.639	
Lys	0.512	0.541	0.267	1.104	2.888	1.825	
His	0.169	0.179	0.087	0.429	1.345	0.754	
Phe	0.376	0.428	0.225	0.927	3.994	3.278	
Trp	0.107	0.114	0.057	0.233	0.774	0.650	
Thr	0.350	0.370	0.182	0.791	2.227	1.363	
Leu	0.477	0.504	0.247	1.112	4.033	2.297	
Ile	0.369	0.391	0.192	0.816	2.571	1.548	
Val	0.442	0.502	0.266	1.196	5.482	3.261	
Arg	0.434	0.457	0.225	0.972	3.135	1.739	
TEAA	3.416	3.679	1.847	7.985	27.51	17.36	

**Table 8:** Estimated intestinally absorbable essential amino acid flows using regression-estimated Kd values under positive ruminal nitrogen balance (g/kg DM).

**Item**	**Soybean** **meal**	**Wheat**	**DDGS**	**Molassed** **Soybean hulls**	**Extruded** **Soybean hulls**	**Wheat** **middlings**	**Alfalfa**
Met	3.610	1.014	1.029	0.819	0.397	1.055	0.996
Lys	13.52	3.007	2.754	2.497	1.122	3.079	3.050
His	4.897	1.034	1.375	0.837	0.371	1.118	1.010
Phe	11.16	2.371	4.953	1.998	1.091	2.558	2.277
Trp	3.112	0.586	0.633	0.546	0.489	0.678	0.625
Thr	9.022	2.013	2.275	1.650	0.701	2.127	2.087
Leu	13.63	2.732	4.821	2.267	0.974	2.998	2.877
Ile	9.847	2.105	2.403	1.733	0.744	2.241	2.201
Val	12.23	2.776	4.585	2.383	1.260	3.099	2.796
Arg	12.18	2.444	2.706	2.049	0.867	2.801	2.558
TEAA	93.20	20.08	27.54	16.78	8.015	21.75	20.48
**Item**	**Wheat** **bran**	**Flaked** **corn**	**Cottonseed 1**	**Cottonseed 2**	**Pelleted sugar** **beet pulp**	**Whole-plant** **corn silage 1**	
Met	1.131	0.451	0.816	0.718	0.627	0.114	
Lys	3.299	1.162	2.148	2.108	1.849	0.296	
His	1.214	0.429	0.958	0.926	0.631	0.099	
Phe	2.636	1.185	2.873	4.047	1.551	0.267	
Trp	0.687	0.263	0.537	0.539	0.354	0.066	
Thr	2.247	0.826	1.494	1.483	1.232	0.203	
Leu	3.190	1.280	2.174	2.238	1.647	0.281	
Ile	2.398	0.859	1.538	1.547	1.267	0.215	
Val	3.221	1.387	3.792	3.762	1.959	0.309	
Arg	3.031	1.011	2.909	2.867	1.451	0.250	
TEAA	23.06	8.853	19.24	20.23	12.57	2.100	
**Item**	**Whole-plant** **corn silage 2**	**Corn** **stover silage**	**Wheat** **straw**	**Dried** **brewer grains**	**Dried distiller** **grains 1**	**Dried distiller** **grains 2**	
Met	0.178	0.180	0.101	0.398	0.911	0.677	
Lys	0.511	0.491	0.274	1.098	2.532	1.917	
His	0.169	0.163	0.089	0.420	1.125	0.811	
Phe	0.374	0.412	0.225	0.900	3.202	3.703	
Trp	0.107	0.105	0.058	0.230	0.651	0.718	
Thr	0.350	0.336	0.187	0.784	1.917	1.445	
Leu	0.476	0.460	0.253	1.097	3.345	2.489	
Ile	0.368	0.355	0.196	0.809	2.186	1.655	
Val	0.440	0.481	0.266	1.155	4.349	3.655	
Arg	0.434	0.415	0.231	0.964	2.654	1.849	
TEAA	3.407	3.398	1.880	7.855	22.87	18.92	

Under the condition of ruminal nitrogen deficiency:

**Table 9:** Estimated intestinally absorbable essential amino acid flows using in situ-fitted Kd values under ruminal nitrogen deficiency (g/kg DM).

**Item**	**Soybean meal**	**Wheat**	**DDGS**	**Molassed** **Soybean hulls**	**Extruded** **Soybean hulls**	**Wheat** **middlings**	**Alfalfa**
Met	1.182	0.189	0.673	0.161	0.105	0.172	0.113
Lys	7.733	0.466	1.472	0.480	0.244	0.377	0.349
His	3.303	0.208	1.187	0.180	0.082	0.233	0.123
Phe	9.272	0.854	5.734	0.847	0.475	0.865	0.558
Trp	2.241	0.079	0.420	0.159	0.270	0.142	0.087
Thr	4.945	0.275	1.598	0.261	0.113	0.286	0.246
Leu	9.150	0.401	4.781	0.415	0.179	0.525	0.399
Ile	5.796	0.274	1.694	0.272	0.124	0.304	0.264
Val	9.249	0.955	4.862	1.007	0.532	1.079	0.739
Arg	7.728	0.269	1.799	0.318	0.136	0.510	0.268
TEAA	60.60	3.972	24.22	4.100	2.262	4.493	3.146
**Item**	**Wheat** **bran**	**Flaked** **corn**	**Cottonseed 1**	**Cottonseed 2**	**Pelleted sugar** **beet pulp**	**Whole-plant** **corn silage 1**	
Met	0.194	0.154	0.349	0.309	0.230	0.059	
Lys	0.399	0.158	0.759	0.859	0.594	0.139	
His	0.290	0.121	0.474	0.509	0.233	0.047	
Phe	0.925	0.756	1.829	3.176	0.990	0.150	
Trp	0.117	0.078	0.248	0.287	0.096	0.033	
Thr	0.270	0.156	0.543	0.630	0.360	0.096	
Leu	0.568	0.456	0.878	1.084	0.470	0.135	
Ile	0.325	0.150	0.542	0.650	0.332	0.101	
Val	1.193	0.862	2.506	2.755	1.353	0.171	
Arg	0.616	0.170	1.609	1.773	0.317	0.117	
TEAA	4.896	3.060	9.737	12.03	4.977	1.049	
**Item**	**Whole-plant** **corn silage 2**	**Corn** **stover silage**	**Wheat** **straw**	**Dried** **brewer grains**	**Dried distiller** **grains 1**	**Dried distiller** **grains 2**	
Met	0.055	0.061	0.041	0.174	0.844	0.354	
Lys	0.134	0.136	0.083	0.370	2.110	0.883	
His	0.045	0.046	0.026	0.203	1.239	0.527	
Phe	0.140	0.173	0.119	0.517	4.321	3.812	
Trp	0.032	0.033	0.021	0.090	0.694	0.615	
Thr	0.093	0.094	0.057	0.300	1.792	0.782	
Leu	0.131	0.133	0.078	0.463	3.840	1.765	
Ile	0.098	0.100	0.059	0.295	2.201	0.999	
Val	0.160	0.197	0.139	0.726	6.139	3.553	
Arg	0.113	0.114	0.068	0.357	2.730	1.039	
TEAA	1.001	1.087	0.693	3.494	25.909	14.33	

**Table 10:** Estimated intestinally absorbable essential amino acid flows using regression-estimated Kd values under ruminal nitrogen deficiency (g/kg DM).

**Item**	**Soybean** **meal**	**Wheat**	**DDGS**	**Molassed** **Soybean hulls**	**Extruded** **Soybean hulls**	**Wheat** **middlings**	**Alfalfa**
Met	1.275	0.199	0.628	0.165	0.134	0.235	0.149
Lys	7.999	0.507	1.432	0.496	0.313	0.541	0.458
His	3.386	0.217	1.058	0.182	0.105	0.309	0.160
Phe	9.420	0.831	4.917	0.787	0.599	1.092	0.677
Trp	2.291	0.088	0.391	0.154	0.339	0.187	0.112
Thr	5.128	0.306	1.472	0.281	0.147	0.403	0.321
Leu	9.384	0.440	4.179	0.433	0.231	0.711	0.511
Ile	5.985	0.308	1.560	0.292	0.161	0.428	0.344
Val	9.433	0.933	4.214	0.937	0.670	1.359	0.893
Arg	7.948	0.314	1.675	0.343	0.177	0.686	0.356
TEAA	62.25	4.144	21.53	4.071	2.877	5.952	3.982
**Item**	**Wheat** **Bran**	**Flaked Corn**	**Cottonseed 1**	**Cottonseed 2**	**Pelleted Sugar** **beet pulp**	**Whole-plant** **corn silage 1**	
Met	0.219	0.126	0.435	0.384	0.176	0.064	
Lys	0.482	0.163	0.956	1.075	0.465	0.144	
His	0.311	0.103	0.587	0.618	0.178	0.049	
Phe	0.951	0.569	2.240	3.762	0.702	0.173	
Trp	0.132	0.065	0.308	0.350	0.078	0.036	
Thr	0.326	0.146	0.683	0.785	0.288	0.100	
Leu	0.636	0.371	1.097	1.332	0.377	0.142	
Ile	0.383	0.143	0.683	0.811	0.271	0.106	
Val	1.221	0.650	3.065	3.278	0.951	0.197	
Arg	0.676	0.164	1.981	2.135	0.271	0.121	
TEAA	5.336	2.501	12.04	14.53	3.757	1.133	
**Item**	**Whole-plant** **corn silage 2**	**Corn** **stover silage**	**Wheat** **straw**	**Dried** **brewer grains**	**Dried distiller** **grains 1**	**Dried distiller** **grains 2**	
Met	0.057	0.069	0.043	0.180	0.640	0.405	
Lys	0.140	0.147	0.091	0.400	1.640	1.027	
His	0.047	0.050	0.029	0.207	0.908	0.587	
Phe	0.143	0.200	0.118	0.517	3.058	4.084	
Trp	0.033	0.037	0.022	0.095	0.512	0.668	
Thr	0.097	0.101	0.062	0.317	1.358	0.891	
Leu	0.136	0.145	0.086	0.482	2.794	1.950	
Ile	0.102	0.108	0.065	0.315	1.641	1.123	
Val	0.163	0.227	0.138	0.717	4.315	3.822	
Arg	0.119	0.122	0.075	0.380	2.028	1.177	
TEAA	1.038	1.206	0.730	3.611	18.89	15.74	

## 4. Discussion

### 4.1. Overall Contribution and Scope of the Study

This study integrated measured chemical composition, CNCPS fractions, in situ-fitted degradation constants, insoluble-protein amino acid composition, and model-derived amino acid flow estimates for the same feed samples. This common framework enabled internally consistent comparisons without combining data from unrelated sources or protocols. However, because only 19 feedstuffs and one representative in situ composite per feedstuff were evaluated, the results characterize the tested samples rather than provide population reference values or universally applicable model inputs.

### 4.2. Evaluation of Carbohydrate and Protein Fractions in Common Feeds for Ruminants

#### 4.2.1. Conventional Nutrient Composition

Ensiled grasses and legumes generally contain more NPN (30–65% of total N) than hay (15–25%) [30]. In the present silages, NPN accounted for 93.82–96.80% of SCP, indicating that most soluble CP was present as NPN, consistent with previous findings [31]. Proteolysis during ensiling converts true protein to NPN, while ammonia N is an indicator of silage protein quality [32]. Differences between the two whole-plant corn silages may reflect harvest maturity, which affects forage quality [33], intake, and digestibility [34], as well as differences in origin and processing. Among roughages, alfalfa combined relatively high CP with low ADIP, whereas the high EE content of cottonseed reflected its energy–protein characteristics.

#### 4.2.2. Carbohydrate Fractions

The greater starch and CNSC contents of energy feeds indicate a greater supply of rapidly fermentable carbohydrate for microbial growth and energy production. In contrast, roughages contained more structural carbohydrate and less CNSC. Their CC proportions varied with forage species, tissue structure, and lignification, which determine cell-wall availability and feed energy value [35]. Thus, carbohydrate fractionation provides information not conveyed by total CHO or fiber concentrations alone.

#### 4.2.3. Protein Fractions

Protein solubility and tissue structure influence degradability [36]. CNCPS separates protein into PA, the true-protein fractions PB1–PB3, and unavailable PC [37]; their distribution affects ruminal degradation and intestinal digestibility [38]. The high PA proportions of the silages likely resulted from proteolysis and NPN formation during ensiling [39], whereas greater PC may reduce intestinal protein availability [39]. Soybean meal had low PA and the highest PB2 proportion, and processing may similarly shift protein from PA toward PB2, as reported for roasted versus raw soybeans [40]. Feed protein value should therefore be evaluated from both CP concentration and fraction distribution.

#### 4.2.4. Relationships Between Conventional Nutrients and CNCPS Fractions

Some CNCPS fractions were partly associated with conventional nutrients. PC was related to CP and ash, consistent with reported associations of PA and PC with routine chemical indicators [41,42], whereas soluble-protein fractions were weakly explained by conventional composition alone.

In the present calculations, CA represented rapidly fermentable non-starch carbohydrate estimated by difference, CB1 represented starch, CB2 represented potentially available cell-wall carbohydrate, and CC represented unavailable cell-wall carbohydrate. The observed relationships largely reflected these definitions: CA was associated with CHO and ADF; CB1 decreased as NDF and ADF increased; CB2 was related to NDF and inversely related to ADF; and CC was associated with ADF. CNSC was excluded because of its mathematical dependence on other carbohydrate variables.

Because only 19 nutritionally diverse feedstuffs were evaluated, these equations may partly reflect feed-category contrasts and should be regarded as hypothesis-generating rather than generalizable relationships.

### 4.3. Ruminal Degradation Kinetics of Carbohydrate and Protein Fractions

#### 4.3.1. Protein-Fraction Degradation Constants

PB1 is expected to degrade faster than PB2 and PB3. The reported CNCPS ranges are 3–16%/h for PB2 and 0.06–0.55%/h for PB3 [6]. The fitted Kd2 values largely overlapped the PB2 range, whereas all Kd3 values exceeded the PB3 range. Kd3 should therefore be interpreted as a parameter inferred from total corrected CP disappearance rather than as direct confirmation of the canonical PB3 rate.

Kd1, Kd2, and Kd3 were estimated jointly rather than from separately measured fraction disappearance. Consequently, Kd1 values above 100%/h are mathematically possible and indicate extremely rapid apparent PB1 disappearance, not degradation of more than 100% within 1 h. Rapid solubilization and physical loss of fine particles through the 50 μm bags during incubation and washing may have contributed to these values [43]. Thus, the highest Kd1 estimates should be regarded as fitted constants describing the rapid early disappearance curve and interpreted cautiously.

#### 4.3.2. Carbohydrate-Fraction Degradation Constants

As shown in Table 5, four carbohydrate-fraction degradation curves had R^2^ values below 0.50: the Kd4 curves of whole-plant corn silages 1 and 2 and the Kd6 curves of wheat and cottonseed 2. The poor Kd4 fits may reflect substantial CA disappearance before the first 2 h sampling point, whereas the poor Kd6 fits may reflect limited or irregular CB2 disappearance, particle loss, and analytical variation. Therefore, these four fitted rate constants should not be used for quantitative comparisons among feedstuffs.

Differences in Kd5 likely reflected starch source, processing, particle size, and bag losses. In situ methods may overestimate rapid starch disappearance through loss of fine or soluble material [44]. Grain starch structure also differs among sources [45], while processing increases microbial accessibility [46] and can alter corn starch degradation through changes in particle size and physical form [47]. Kd5 should therefore be interpreted with feed source and processing characteristics.

Fiber degradation depends on cell-wall structure, lignification, feed type, and ruminal conditions rather than NDF concentration alone. Greater lignification may restrict roughage fiber utilization [48], and differences between the two whole-plant corn silages may partly reflect maturity-related reductions in fiber degradability [11].

#### 4.3.3. Regression Relationships Between Conventional Nutrients, Feed Fractions, and Ruminal Degradation Rate Constants of Protein Fractions

The coefficient directions were generally biologically plausible. Kd1 was positively associated with PB1 and negatively associated with NDIP and CC, whereas Kd2 was positively associated with CA and ash and negatively associated with ADF. These patterns may reflect synchronization between readily fermentable carbohydrate and nitrogen degradation: microbial protein associated with CNSC is derived from both peptides and ammonia N [49], whereas CC and ADF provide little or slowly available energy. Kd3 was positively related to starch, but its low R^2^ indicates that starch explained only a limited proportion of PB3 degradation-rate variation. Protein matrix structure, heat processing, fiber association, particle size, and ruminal conditions may also affect this slowly degradable fraction.

Diagnostics supported the relative stability of the Kd1 and Kd2 equations: multicollinearity was low, residuals did not significantly depart from normality, and coefficient directions were retained in sensitivity analyses. However, the significance of CC in the Kd1 equation was sensitive to cottonseed 1. Kd3 was less robust, with non-normal residuals, lower explanatory power, and greater sensitivity to individual feedstuffs. Leave-one-observation-out analyses assessed coefficient sensitivity but not out-of-sample error; therefore, none of the equations has demonstrated predictive performance for untested feeds.

Residuals did not differ significantly between broad concentrate-type and roughage groups, but the small, unbalanced category sizes limited statistical power. Methodological uncertainty also remains: zero-hour bags quantified washing and immediate particle losses, blank bags monitored background changes, and residual N was corrected for microbial contamination, yet fine-particle loss through the 50 μm pores could not be eliminated. Furthermore, because each feedstuff was evaluated in a separate run, run-to-run variation could not be distinguished fully from feedstuff differences. Because the nonlinear degradation curves were fitted to feedstuff-level mean disappearance values across the four cows, between-cow variability in the fitted Kd constants could not be quantified. Accordingly, the reported Kd values should be interpreted as feedstuff-level descriptive estimates rather than replicated animal-level parameters.

### 4.4. Interpretation of CNCPS-Based Intestinally Absorbable Amino Acid Flow Estimates

#### 4.4.1. Insoluble-Protein Amino Acid Composition

The amino acid profile of rumen-undegraded protein may differ from total dietary protein but resembles that of feed insoluble protein [12], supporting its use as a proxy in the present calculations. Insoluble-protein amino acid profiles varied among feedstuffs, indicating that a single fixed profile is inadequate. The measured concentrations were lower than values summarized by Edmunds et al. [50] but generally consistent with feed-fraction profiles reported by Zhang [51]. High PA reduces the contribution of rumen-undegraded feed protein, whereas ADIP may restrict intestinal amino acid availability, although some ADIP-associated amino acids in concentrates may remain available [13]. Both amino acid composition and protein-fraction distribution should therefore be considered.

#### 4.4.2. Amino Acid Flow Estimates from Alternative Kd Inputs

The two amino acid-flow datasets were generated within the same CNCPS framework using identical feed composition, amino acid profiles, CNCPS fractions, Kp inputs, and nitrogen-balance scenarios; only the source of Kd differed. Their agreement therefore does not independently validate either the regressions or the flow estimates. Feed-derived amino acid flow depends on passage relative to degradation of PB1–PB3 [52] and is particularly sensitive to PB2 degradation rate [53].

Comparisons among protein-evaluation models have reported broadly similar flow estimates [54], although other work found CNCPS more accurate than NRC for absorbable essential amino acids [55]. Total intestinal supply includes microbial, rumen-undegraded feed, and endogenous protein [56]. Moreover, postruminal amino acid and energy supplies can independently affect performance and metabolism in dairy cows [57], supporting the nutritional relevance of estimating these flows.

Ruminal nitrogen deficiency may reduce microbial protein synthesis and fiber degradation [58], while degradable protein and starch supplementation can improve low-quality forage utilization [59]. A dietary CP concentration of approximately 6–8% has been proposed as the minimum required to support ruminal microbial activity in low-quality forage diets [37]. In this study, however, nitrogen balance was neither measured nor experimentally manipulated. Positive balance and deficiency were alternative CNCPS scenarios; their differences represent model responses rather than observed physiological effects.

Kp was also model-derived. Standardized inputs of 4% BW for DMI and 50% dietary concentrate differed from the donor-cow means of 5.13% BW and 58.38% concentrate. Calculated Kp values were 5.5%/h for wet forages, 4.3%/h for wheat straw, 4.9%/h for alfalfa, and 7.4%/h for concentrate-type feeds. The NRC equations do not fully account for particle characteristics, processing, liquid flow, or temporal intake variation [26]; therefore, the absolute amino acid flows apply only to the specified scenario and require recalculation for other animals or diets.

Finally, although each feedstuff was represented by multiple batches, the samples covered limited commercial sources and regions. The regression dataset included only 19 feedstuffs, lacked external validation, and contained small, unbalanced feed categories. The equations should therefore be regarded as sample-dependent exploratory associations requiring evaluation in larger, category-balanced, independent datasets.

## 5. Conclusions

This study integrated chemical composition, CNCPS fractions, in situ degradation kinetics, insoluble-protein amino acid profiles, and model-derived intestinal amino acid flows for 19 dairy feedstuffs. The Kd1 and Kd2 regressions showed stronger in-sample relationships than Kd3, but all equations were exploratory, sample-dependent, and require validation with independent feeds before application; Kd3 should not be used for individual-feed prediction. Amino acid flows calculated using in situ-fitted and regression-estimated Kd values were generally comparable, but both were CNCPS-derived estimates rather than direct measurements, and their agreement did not constitute validation. Because the estimates depended on NRC-derived Kp values under a standardized dietary scenario, they should not be generalized to other diets, intake levels, or animals without recalculation.

## Figures and Tables

**Table 1:** Conventional chemical composition of the tested feed samples.

Feed	DM	CP	NDF	ADF	EE	Ash	ADL	Starch
		DM	DM	DM	DM	DM	DM	DM
Soybean meal	87.79 ± 0.55	49.45 ± 1.11	15.61 ± 1.12	10.25 ± 0.74	1.46 ± 0.13	6.59 ± 0.36	0.18 ± 0.02	8.34 ± 0.90
Wheat	87.18 ± 0.70	15.60 ± 0.62	19.16 ± 1.92	2.46 ± 0.30	2.00 ± 0.20	3.22 ± 0.19	0.46 ± 0.07	58.32 ± 2.33
DDGS	89.32 ± 0.98	32.74 ± 1.80	39.11 ± 3.01	10.38 ± 0.91	5.31 ± 0.41	4.72 ± 0.36	3.15 ± 0.35	12.20 ± 1.34
Molassed soybean hulls	91.11 ± 0.77	15.74 ± 0.66	56.13 ± 2.95	33.66 ± 2.12	4.52 ± 0.38	4.86 ± 0.31	1.86 ± 0.20	5.66 ± 0.71
Extruded soybean hulls	89.82 ± 0.68	11.33 ± 0.43	70.45 ± 3.35	45.41 ± 2.59	1.39 ± 0.11	4.93 ± 0.28	1.71 ± 0.16	3.51 ± 0.40
Wheat middlings	88.46 ± 0.97	20.73 ± 1.14	26.92 ± 2.96	5.62 ± 0.74	4.47 ± 0.39	3.27 ± 0.29	1.24 ± 0.16	40.39 ± 3.55
Wheat bran	86.73 ± 0.91	19.65 ± 1.03	47.70 ± 5.01	11.60 ± 1.46	6.98 ± 0.59	7.04 ± 0.59	3.03 ± 0.38	11.21 ± 0.94
Flaked corn	86.65 ± 0.62	9.22 ± 0.33	17.78 ± 1.60	3.37 ± 0.36	3.36 ± 0.30	1.29 ± 0.07	0.74 ± 0.10	64.29 ± 2.31
Cottonseed 1	89.68 ± 0.90	23.58 ± 0.94	45.83 ± 2.75	40.88 ± 2.86	12.50 ± 0.75	3.91 ± 0.27	15.86 ± 1.27	5.40 ± 0.65
Cottonseed 2	92.45 ± 1.02	24.78 ± 1.09	41.15 ± 2.72	32.58 ± 2.51	19.72 ± 1.30	4.58 ± 0.35	11.98 ± 1.05	5.92 ± 0.78
Pelleted sugar beet pulp	91.48 ± 0.70	12.40 ± 0.47	50.03 ± 2.38	25.28 ± 1.44	4.05 ± 0.31	4.19 ± 0.24	2.55 ± 0.24	5.57 ± 0.63
whole-plant corn silage 1	29.93 ± 1.16	9.37 ± 0.59	67.73 ± 4.98	30.23 ± 2.54	3.87 ± 0.41	5.28 ± 0.44	7.62 ± 0.80	6.02 ± 0.76
whole-plant corn silage 2	35.10 ± 1.30	8.69 ± 0.57	36.88 ± 2.84	21.56 ± 1.90	4.07 ± 0.45	4.73 ± 0.42	2.73 ± 0.30	7.38 ± 0.97
Corn stover silage	24.93 ± 1.15	9.22 ± 0.61	60.96 ± 4.69	35.74 ± 3.15	5.45 ± 0.60	8.38 ± 0.74	4.39 ± 0.48	5.51 ± 0.73
Wheat straw	90.46 ± 0.90	4.98 ± 0.33	78.65 ± 6.06	51.83 ± 4.56	1.31 ± 0.14	13.41 ± 1.18	7.89 ± 0.87	2.82 ± 0.47
Alfalfa	91.68 ± 0.83	16.32 ± 0.98	44.55 ± 3.12	34.69 ± 2.78	3.60 ± 0.36	3.52 ± 0.28	7.70 ± 0.77	5.08 ± 0.76
Dried brewer grains	93.83 ± 1.03	24.76 ± 1.36	52.79 ± 4.06	23.86 ± 2.10	12.94 ± 1.00	4.25 ± 0.33	6.58 ± 0.72	2.27 ± 0.25
Dried distiller grains 1	93.60 ± 0.98	30.33 ± 1.59	49.33 ± 3.63	21.35 ± 1.79	10.87 ± 0.80	4.39 ± 0.32	4.17 ± 0.44	5.31 ± 0.56
Dried distiller grains 2	92.80 ± 1.02	23.54 ± 1.29	53.01 ± 4.08	19.89 ± 1.75	10.01 ± 0.77	4.68 ± 0.36	4.00 ± 0.44	6.91 ± 0.76

Values are presented as means ± SD of independent batch-level values (*n* ≥ 3). Each independent batch composite was analyzed in triplicate, and the analytical replicates were averaged to obtain one batch-level value. DM is expressed as % as-fed; CP, NDF, ADF, EE, ash, ADL, and starch are expressed as % DM. For silage samples, the reported DM values were determined using the original fresh samples before freeze-drying.

**Table 2:** Carbohydrate fractions of the tested feed samples.

Feed	CHO	CA	CB1	CNSC	CB2	CC
	DM	CHO	CHO	CHO	CHO	CHO
Soybean meal	42.50 ± 2.42	46.84 ± 0.91	19.62 ± 0.15	66.46 ± 2.76	32.51 ± 0.95	1.03 ± 0.03
Wheat	79.18 ± 0.35	9.26 ± 0.98	73.65 ± 0.55	82.91 ± 2.56	15.70 ± 0.90	1.39 ± 0.10
DDGS	57.24 ± 0.26	19.41 ± 0.36	21.32 ± 0.35	40.73 ± 1.98	46.08 ± 0.02	13.19 ± 0.74
Molassed soybean hulls	74.88 ± 0.48	20.92 ± 0.67	7.55 ± 0.43	28.48 ± 0.24	65.57 ± 0.03	5.95 ± 0.62
Extruded soybean hulls	82.35 ± 0.45	16.44 ± 0.30	4.26 ± 0.12	20.70 ± 0.18	74.32 ± 0.83	4.98 ± 0.44
Wheat middlings	71.54 ± 0.66	8.15 ± 0.69	56.46 ± 0.47	64.61 ± 1.22	31.21 ± 0.55	4.17 ± 0.07
Wheat bran	66.33 ± 0.40	20.48 ± 0.69	16.90 ± 0.24	37.38 ± 0.44	51.65 ± 0.54	10.97 ± 1.01
Flaked corn	86.13 ± 1.13	6.97 ± 0.78	74.65 ± 0.35	81.61 ± 1.43	16.33 ± 0.35	2.06 ± 0.57
Cottonseed 1	60.01 ± 2.66	18.75 ± 0.49	8.99 ± 0.06	27.74 ± 0.56	8.85 ± 0.21	63.41 ± 0.71
Cottonseed 2	50.92 ± 0.98	16.56 ± 0.31	11.62 ± 0.18	28.18 ± 1.49	15.33 ± 0.61	56.49 ± 0.24
Pelleted sugar beet pulp	79.36 ± 1.16	36.61 ± 0.55	7.02 ± 0.33	43.64 ± 0.22	48.66 ± 0.01	7.70 ± 0.42
whole-plant corn silage 1	81.48 ± 0.36	12.03 ± 0.95	7.39 ± 0.67	19.42 ± 0.72	58.14 ± 0.14	22.44 ± 0.13
whole-plant corn silage 2	82.51 ± 0.68	47.82 ± 0.19	8.95 ± 0.15	56.77 ± 0.04	35.30 ± 0.11	7.93 ± 0.34
Corn stover silage	76.95 ± 0.53	15.21 ± 0.26	7.16 ± 0.02	22.37 ± 1.29	63.94 ± 0.23	13.69 ± 0.96
Wheat straw	80.30 ± 0.76	0.06 ± 0.02	3.51 ± 0.07	3.57 ± 0.91	72.84 ± 0.17	23.59 ± 0.74
Alfalfa	76.56 ± 0.07	36.55 ± 0.04	6.64 ± 0.48	43.19 ± 0.44	32.67 ± 0.75	24.14 ± 0.35
Dried brewer grains	58.05 ± 0.01	8.18 ± 0.72	3.91 ± 0.13	12.09 ± 0.59	60.72 ± 0.82	27.19 ± 0.14
Dried distiller grains 1	54.41 ± 1.14	7.82 ± 0.63	9.76 ± 0.01	17.58 ± 0.62	64.04 ± 0.57	18.38 ± 0.24
Dried distiller grains 2	61.77 ± 0.83	9.51 ± 0.79	11.18 ± 0.66	20.69 ± 0.87	63.77 ± 0.54	15.53 ± 0.26

Values are presented as means ± SD of independent batch-level values (*n* ≥ 3). CHO is expressed as % DM; CA, CB1, CNSC, CB2, and CC are expressed as % CHO. CNSC, combined non-structural carbohydrate fraction calculated as the sum of CA and CB1 (CNSC = CA + CB1).

**Table 3:** Protein fractions of the tested feed samples (% CP).

Feed	PA	PB1	PB2	PB3	PC
Soybean meal	7.81 ± 0.86	6.78 ± 0.62	83.01 ± 0.48	0.46 ± 0.09	1.94 ± 0.02
Wheat	8.67 ± 0.16	24.40 ± 0.11	35.52 ± 0.01	25.52 ± 0.18	5.89 ± 0.11
DDGS	22.84 ± 0.25	3.07 ± 0.03	59.95 ± 1.83	11.31 ± 0.31	2.83 ± 0.02
Molassed soybean hulls	15.85 ± 0.28	18.19 ± 0.61	51.78 ± 0.43	8.23 ± 0.12	5.95 ± 0.03
Extruded soybean hulls	15.20 ± 0.25	9.02 ± 0.36	61.66 ± 0.73	9.44 ± 0.42	4.68 ± 0.05
Wheat middlings	14.59 ± 0.04	25.31 ± 0.51	53.41 ± 0.59	4.49 ± 0.10	2.21 ± 0.01
Wheat bran	15.13 ± 0.17	18.69 ± 0.54	38.03 ± 0.91	23.33 ± 0.03	4.81 ± 0.07
Flaked corn	9.28 ± 0.26	1.47 ± 0.01	69.73 ± 0.28	9.00 ± 0.06	10.47 ± 0.06
Cottonseed 1	23.81 ± 0.96	4.89 ± 0.68	61.75 ± 0.30	5.89 ± 0.01	3.66 ± 0.24
Cottonseed 2	12.44 ± 0.13	5.62 ± 0.05	64.43 ± 0.08	14.41 ± 0.51	3.11 ± 0.03
Pelleted sugar beet pulp	12.69 ± 0.03	0.08 ± 0.01	48.20 ± 0.12	35.62 ± 0.50	3.47 ± 0.50
whole-plant corn silage 1	69.55 ± 0.03	2.46 ± 0.04	7.98 ± 0.01	9.28 ± 0.07	10.73 ± 0.07
whole-plant corn silage 2	63.35 ± 0.16	4.17 ± 0.11	19.69 ± 0.04	1.37 ± 0.14	11.41 ± 0.14
Corn stover silage	61.29 ± 0.02	2.02 ± 0.03	24.20 ± 0.01	1.59 ± 0.15	10.90 ± 0.11
Wheat straw	55.06 ± 0.26	8.03 ± 0.08	14.02 ± 0.03	4.35 ± 0.01	18.54 ± 0.02
Alfalfa	17.59 ± 0.55	12.40 ± 0.54	59.29 ± 0.21	4.84 ± 0.01	5.88 ± 0.04
Dried brewer grains	73.45 ± 0.28	7.72 ± 0.12	12.35 ± 0.56	2.92 ± 0.02	3.57 ± 0.02
Dried distiller grains 1	4.79 ± 0.06	2.49 ± 0.02	78.88 ± 0.69	10.58 ± 0.02	3.27 ± 0.06
Dried distiller grains 2	3.14 ± 0.13	0.76 ± 0.01	80.24 ± 0.21	11.67 ± 0.07	4.19 ± 0.06

Values are presented as means ± SD of independent batch-level values (*n* ≥ 3). PA, PB1, PB2, PB3, and PC are expressed as % CP. NDIP and ADIP were originally expressed on a DM basis; values were converted to a CP basis before regression analysis. PA, PB1, PB2, PB3, and PC summed to 100% of CP before rounding; minor deviations in the displayed totals (99.95–100.06%) resulted from rounding individual values to two decimal places.

**Table 4:** In situ-fitted ruminal degradation rate constants of protein fractions in the tested feed samples.

Feed	Kd1 (%/h)	Kd2 (%/h)	Kd3 (%/h)	R^2^
Soybean meal	252.90	18.73	1.78	0.839
Wheat	93.79	9.41	6.74	0.762
DDGS	159.50	6.15	2.03	0.798
Molassed soybean hulls	236.70	5.09	2.54	0.970
Extruded soybean hulls	145.10	2.29	1.85	0.777
Wheat middlings	250.20	8.05	7.19	0.893
Wheat bran	396.20	14.91	5.92	0.805
Flaked corn	122.40	8.40	8.16	0.822
Cottonseed 1	37.05	2.66	2.25	0.760
Cottonseed 2	46.93	8.47	6.76	0.887
Pelleted sugar beet pulp	177.50	11.96	2.45	0.890
whole-plant corn silage 1	137.50	9.41	6.92	0.862
whole-plant corn silage 2	177.00	14.31	6.60	0.978
Corn stover silage	88.71	11.79	1.62	0.646
Wheat straw	86.42	5.21	4.47	0.815
Alfalfa	214.50	12.28	1.35	0.796
Dried brewer grains	101.80	4.73	2.05	0.884
Dried distiller grains 1	145.60	2.98	2.07	0.687
Dried distiller grains 2	113.40	9.42	4.48	0.870

Kd1, Kd2, and Kd3 are the ruminal degradation rate constants of protein fractions PB1, PB2, and PB3, respectively. R^2^ is the coefficient of determination for the feedstuff-level nonlinear fit between the model-predicted and observed mean corrected CP disappearance values across incubation times. Values are feedstuff-level rate constants obtained by fitting the mean corrected CP disappearance values of the four cows at each incubation time. Kd1 values greater than 100%/h represent fitted first-order rate constants and should not be interpreted as directly observed percentages of PB1 degraded within 1 h.

**Table 5:** In situ-fitted ruminal degradation rate constants of carbohydrate fractions in the tested feed samples.

Feed	Kd4 (%/h)	R^2^	Kd5 (%/h)	R^2^	Kd6 (%/h)	R^2^
Soybean meal	56.49	0.999	47.18	0.977	1.739	0.767
Wheat	128.9	0.996	13.84	0.941	0.2865	0.030 ^+^
DDGS	118.6	0.996	2.757	0.979	2.291	0.956
Molassed soybean hulls	60.30	0.967	5.108	0.950	1.836	0.955
Extruded soybean hulls	32.76	0.992	5.331	0.975	1.293	0.852
Wheat middlings	28.63	0.988	16.45	0.926	2.413	0.921
Wheat bran	101.1	0.540	8.395	0.920	1.875	0.863
Flaked corn	214.6	0.722	5.731	0.994	2.034	0.914
Cottonseed 1	16.89	0.949	1.004	0.745	2.955	0.869
Cottonseed 2	20.13	0.831	2.592	0.949	1.929	0.269 ^+^
Pelleted sugar beet pulp	26.67	0.716	3.854	0.953	4.513	0.930
whole-plant corn silage 1	28.40	0.442 ^+^	26.48	0.841	1.175	0.647
whole-plant corn silage 2	32.00	0.498 ^+^	18.55	0.876	1.872	0.680
Corn stover silage	150.8	0.936	40.06	0.936	2.172	0.994
Wheat straw	50.04	0.941	2.676	0.907	1.51	0.955
Alfalfa	32.49	0.761	10.29	0.838	1.529	0.970
Dried brewer grains	28.82	0.724	9.032	0.834	1.874	0.778
Dried distiller grains 1	34.52	0.714	11.69	0.878	1.773	0.789
Dried distiller grains 2	3.160	0.996	2.124	0.984	1.762	0.952

R^2^ is the coefficient of determination for the corresponding feedstuff-level nonlinear fit to the mean disappearance series. Values are feedstuff-level rate constants obtained by fitting the mean carbohydrate-fraction disappearance values of the four cows at each incubation time. ^+^ Low-fit curve (R^2^ < 0.50); the corresponding fitted degradation rate constant should be regarded as an unreliable estimate and should not be used for quantitative comparisons among feedstuffs.

**Table 6:** Key essential amino acid composition of insoluble protein in the tested feed samples (g/100 g insoluble protein).

Feed	Lys	Met	Total EAA
Soybean meal	2.911	0.261	24.33
Wheat	0.792	0.333	7.065
DDGS	0.719	0.366	14.71
Molassed soybean hulls	0.649	0.218	5.795
Extruded soybean hulls	0.533	0.249	5.500
Wheat middlings	0.486	0.242	6.645
Wheat bran	0.505	0.267	6.983
Flaked corn	0.166	0.247	4.906
Cottonseed 1	0.702	0.344	10.02
Cottonseed 2	0.701	0.256	10.71
Pelleted sugar beet pulp	0.700	0.277	6.047
whole-plant corn silage 1	0.069	0.24	3.087
whole-plant corn silage 2	0.078	0.233	3.159
Corn stover silage	0.109	0.243	3.388
Wheat straw	0.051	0.297	3.494
Alfalfa	0.774	0.25	7.786
Dried brewer grains	0.239	0.279	5.794
Dried distiller grains 1	0.782	0.32	10.186
Dried distiller grains 2	0.648	0.264	11.077

Total EAA represents the sum of Lys, Met, Arg, His, Ile, Leu, Phe, Thr, Trp, and Val. Complete essential and non-essential amino acid profiles are provided in Table S2.

## Data Availability

The original contributions presented in this study are included in the article/Supplementary Materials. Further inquiries can be directed to the corresponding author.

## Supplementary Materials

The following supporting information can be downloaded at: https://www.mdpi.com/article/10.3390/ani16152371/s1, Table S1: Excluded exploratory regression models for CNCPS protein fractions; Table S2: Complete measured amino acid composition of insoluble protein in the tested feed samples (g/100 g insoluble protein).

## Abbreviations

The following abbreviations are used in this manuscript:

DM	Dry matter
CP	Crude protein
NDF	Neutral detergent fiber
ADF	Acid detergent fiber
EE	Ether extract
ADL	Acid detergent lignin
NDIP	Neutral detergent insoluble protein
ADIP	Acid detergent insoluble protein
NPN	Non-protein nitrogen
SCP	Soluble crude protein
CNSC	Non-Structural Carbohydrates
CHO	Total carbohydrate
CA	Rapidly fermentable non-starch carbohydrate fraction
CB1	Starch fraction
CB2	Potentially available cell-wall carbohydrate fraction
CC	Unavailable cell-wall carbohydrate fraction
PA	Non-protein nitrogen fraction
PB1	Rapidly degradable true protein fraction
PB2	Moderately degradable true protein fraction
PB3	Slowly degradable true protein fraction
PC	Unavailable protein fraction
EAA	Essential amino acids
NEAA	Non-essential amino acids
NEL	Net energy for lactation
TAA	Total amino acids
Met	Methionine
Lys	Lysine
His	Histidine
Phe	Phenylalanine
Trp	Tryptophan
Thr	Threonine
Leu	Leucine
Ile	Isoleucine
Val	Valine
Arg	Arginine
TEAA	Total essential amino acids

## Appendix A

**Table A1:** Sampling information, sources, storage conditions, and agronomic or processing characteristics of the tested feed samples.

Feed	Source/ Location	Collection or Purchase Period	Feed Type and Key Agronomic/Processing Characteristics	Storage
Soybean meal	Shandong, China	October–November 2024	Solvent-extracted protein concentrate produced after soybean oil extraction and dried in meal form.	D
Wheat	Shandong, China	June 2024	Mature cereal grain harvested at full maturity, cleaned, dried, and ground before analysis.	D
Flaked corn	Shandong, China	October–November 2024	Mature corn grain steam-conditioned at approximately 95–100 °C for approximately 30 min and roller-flaked to a density of approximately 0.32–0.39 kg/L.	D
Wheat bran	Shandong, China	October–November 2024	Wheat milling by-product consisting primarily of the outer bran fraction.	D
Molassed soybean hulls	Beijing, China	October–November 2024	Soybean hulls mixed with molasses to improve palatability and fermentable carbohydrate supply.	D
Extruded soybean hulls	Beijing, China	October–November 2024	Soybean hulls subjected to extrusion processing and used as a digestible-fiber by-product feed.	D
Pelleted sugar beet pulp	Tianjin, China	October–November 2024	Sugar beet pulp dried and pelleted after sugar extraction.	D
DDGS	Tianjin, China	October–November 2024	Distillers dried grains with solubles produced after grain fermentation, distillation, and drying.	D
Wheat middlings	Tianjin, China	October–November 2024	Wheat milling by-product consisting mainly of fine bran, germ, and residual endosperm particles.	D
Cottonseed 1	Shanxi, China	September–November 2024	Independent whole-cottonseed commercial samples obtained from Shanxi.	D
Cottonseed 2	Tianjin, China	September–November 2024	Independent whole-cottonseed commercial samples obtained from Tianjin.	D
Whole-plant corn silage 1	Beijing Sunlon Livestock Development Co., Ltd., Beijing, China	Harvested September–October 2024; sampled November–December 2024	hole corn plants harvested at the late milk to early dent stage, chopped to approximately 1–2 cm, compacted, and anaerobically ensiled.	S
Whole-plant corn silage 2	Tianjin Jialihe Animal Husbandry Group Co., Ltd., Tianjin, China	Harvested September–October 2024; sampled November–December 2024	Whole corn plants harvested at the half-milk-line to dent stage, chopped to approximately 1–2 cm, compacted, and anaerobically ensiled.	S
Corn stover silage	Tianjin, China	Prepared September–October 2024; sampled November–December 2024	Corn stover collected after grain harvest, chopped, compacted, and anaerobically ensiled	S
Wheat straw	Henan, China	June 2024	Wheat straw collected after grain harvest, air-dried, chopped, and ground before analysis.	D
Alfalfa	Shandong, China	Late May 2024	First-cut alfalfa harvested at the early flowering stage in late May 2024, sun-cured as hay, and subsequently sampled.	D
Dried brewer grains	Henan, China	October–November 2024	Brewer grains generated after mashing and lautering during beer production and subsequently dried.	D
Dried distiller grains 1	Shanxi Chongkang, Shanxi, China	October–November 2024	Independent commercial dried distiller grains obtained from Shanxi Chongkang after grain fermentation and distillation.	D
Dried distiller grains 2	Shanxi Ruimei, Shanxi, China	October–November 2024	Independent commercial dried distiller grains obtained from Shanxi Ruimei after grain fermentation and distillation.	D

Each feed sample was represented by at least three independent production or commercial batches. For each batch, five subsamples were collected from spatially separated locations and combined to form one composite sample. Composite samples from different batches were retained and analyzed separately. Each batch composite was analyzed in triplicate, and the analytical replicates were averaged to obtain one batch-level value. D, dry samples sealed in polyethylene bags and stored at room temperature under cool, dry, and dark conditions; S, silage samples placed in airtight bags immediately after collection, stored at −20 °C, and freeze-dried before analysis. Cottonseed 1 and 2, whole-plant corn silage 1 and 2, and dried distiller grains 1 and 2 were independent sample groups obtained from different production sources and were not biological replicates of the same production batch.

**Table A2:** Ingredient composition and nutrient levels of the lactation-period basal diet fed to ruminally cannulated dairy cows.

Ingredient	Content, % DM	Nutrient Level	Content
Whole-plant corn silage	28.15	NEL, MJ/kg DM	7.11
Alfalfa	13.47	CP, % DM	17.96
Corn meal	11.47	EE, % DM	4.54
Flaked corn	11.77	NDF, % DM	35.88
Soybean meal	15.31	ADF, % DM	21.90
Corn gluten meal	1.68	Ca, % DM	1.03
Double-low rapeseed meal	2.88	P, % DM	0.42
Whole cottonseed	2.93		
Distillers’ grains	6.80		
Palm oil	1.21		
NaHCO_3_	1.35		
Limestone	0.55		
CaHPO_4_	0.52		
MgCl_2_	0.39		
NaCl	0.52		
Premix	1.00		
Total	100.00		

**Table A3:** Nitrogen solubility and detergent-insoluble protein characteristics of the tested feed samples.

Feed	NDIP	ADIP	NPN	SCP
	DM	DM	SCP	CP
Soybean meal	1.35 ± 0.12	1.09 ± 0.10	53.51 ± 3.85	14.59 ± 0.92
Wheat	5.62 ± 0.56	1.05 ± 0.11	26.23 ± 2.62	33.07 ± 2.65
DDGS	5.18 ± 0.46	1.04 ± 0.09	88.14 ± 6.79	25.91 ± 2.85
Molassed soybean hulls	2.57 ± 0.22	1.08 ± 0.11	46.56 ± 3.91	34.04 ± 2.86
Extruded soybean hulls	1.60 ± 0.12	0.53 ± 0.05	62.75 ± 4.77	24.22 ± 1.84
Wheat middlings	6.17 ± 0.68	1.05 ± 0.12	36.57 ± 4.02	39.89 ± 3.51
Wheat bran	1.95 ± 0.20	1.04 ± 0.11	44.74 ± 4.70	33.83 ± 2.84
Flaked corn	2.47 ± 0.22	0.95 ± 0.09	86.34 ± 7.77	10.74 ± 0.77
Cottonseed 1	4.58 ± 0.37	0.81 ± 0.06	82.95 ± 5.81	28.70 ± 2.58
Cottonseed 2	5.30 ± 0.47	0.47 ± 0.04	68.87 ± 5.30	18.06 ± 1.79
Pelleted sugar beet pulp	2.07 ± 0.16	1.11 ± 0.11	99.78 ± 7.58	12.72 ± 0.97
whole-plant corn silage 1	1.21 ± 0.10	1.08 ± 0.09	96.58 ± 2.03	72.01 ± 3.78
whole-plant corn silage 2	1.23 ± 0.11	1.07 ± 0.09	93.82 ± 2.06	67.53 ± 3.71
Corn stover silage	1.22 ± 0.11	0.99 ± 0.09	96.80 ± 2.13	63.32 ± 3.48
Wheat straw	1.06 ± 0.12	1.05 ± 0.12	87.27 ± 7.68	63.09 ± 6.94
Alfalfa	1.75 ± 0.18	0.96 ± 0.10	58.66 ± 4.69	29.99 ± 3.00
Dried brewer grains	4.49 ± 0.40	1.06 ± 0.09	90.49 ± 6.97	81.16 ± 8.93
Dried distiller grains 1	4.02 ± 0.34	1.06 ± 0.09	65.81 ± 4.84	7.27 ± 0.76
Dried distiller grains 2	5.20 ± 0.46	1.09 ± 0.10	80.60 ± 6.21	3.90 ± 0.43

Values are presented as means ± SD of independent batch-level values (*n* ≥ 3). Each independent batch composite was analyzed in triplicate, and the analytical replicates were averaged to obtain one batch-level value. NDIP and ADIP are expressed as % DM; NPN is expressed as % SCP; and SCP is expressed as % CP. NDIP and ADIP are reported on a DM basis in this table; for CNCPS protein-fraction calculations and regression analyses, they were converted to a CP basis as NDIP or ADIP (% CP) = NDIP or ADIP (% DM)/CP (% DM) × 100.

**Table A4:** Multicollinearity, residual-normality, and broad feed-category diagnostics for the exploratory Kd regression models.

Model	Predictor	VIF	Maximum VIF	Shapiro–Wilk W	*p*-Value	Feed-Category Comparison *p*-Value
Kd1	NDIP	1.173	1.260	0.954	0.456	0.150
	PB1	1.260				
	CC	1.087				
Kd2	ADF	1.370	1.385	0.957	0.515	0.159
	Ash	1.385				
	CA	1.015				
Kd3	Starch	1.000	1.000	0.862	0.011	0.419

VIF, variance inflation factor. VIF < 5 was used as the acceptance criterion. Residual normality was evaluated using the Shapiro–Wilk test together with visual inspection of normal Q–Q plots. Feed-category *p*-values represent comparisons of regression residuals between concentrate-type and roughage feedstuffs using Welch’s *t*-test. A *p*-value greater than 0.05 indicated that no statistically significant residual difference was detected between the two broad feed categories.

**Table A5:** Influence diagnostics and leave-one-observation-out sensitivity analyses for the exploratory Kd regression models.

Model	Feedstuff	Cook’s Distance	Leverage	Studentized Residual	R^2^ After Removal	Overall *p*-Value After Removal	Main Finding
Kd1	Wheat	0.826	0.482	−2.085	0.792	<0.001	Coefficient directions unchanged
Kd1	Wheat bran	0.709	0.296	3.376	0.732	<0.001	PB1 coefficient decreased but remained significant
Kd1	Cottonseed 1	0.001	0.461	0.058	0.702	<0.001	CC became marginally nonsignificant, *p* ≈ 0.060
Kd2	Wheat straw	0.447	0.713	-0.841	0.740	<0.001	Coefficient directions and significance unchanged
Kd2	Soybean meal	0.003	0.450	−0.122	0.630	0.003	Model fit decreased; conclusions unchanged
Kd2	DDGS	0.140	0.113	−2.421	0.811	<0.001	Model fit increased; conclusions unchanged
Kd3	Flaked corn	0.001	0.459	0.043	0.261	0.030	Starch coefficient remained positive; R^2^ decreased
Kd3	Wheat	0.097	0.369	−0.563	0.352	0.010	Positive starch association retained
Kd3	Whole-plant corn silage 1	0.115	0.062	2.024	0.468	0.002	Positive starch association retained

Cook’s distance > 4/*n*, leverage > 2(*p* + 1)/*n*, and an absolute studentized residual > 2 were used as screening criteria, where n is the number of feedstuffs and *p* is the number of predictors. With *n* = 19, the Cook’s distance threshold was 0.211. The leverage threshold was 0.421 for the Kd1 and Kd2 models and 0.211 for the Kd3 model. Each model was refitted after removing one identified observation at a time. Observations were not excluded from the final models unless a data-recording or analytical error was identified.

**Table A6:** Summary of analytical procedures used for conventional nutrients and CNCPS-related measurements.

Parameter	Analytical Principle or Procedure	Standard or Methodological Basis
Dry matter	Oven drying to constant weight	GB/T 6435-2014
Crude protein	Kjeldahl determination of total nitrogen; nitrogen concentration multiplied by 6.25	GB/T 6432-2018
Ether extract	Solvent extraction of lipid-soluble components	GB/T 6433-2006
Ash	Combustion in a muffle furnace to remove organic matter	GB/T 6438-2007
Neutral detergent fiber	Neutral-detergent extraction of the insoluble cell-wall residue	GB/T 20806-2022
Acid detergent fiber	Acid-detergent extraction of cellulose, lignin, and acid-insoluble residue	NY/T 1459-2022
Lignin	Sulfuric acid treatment of the acid-detergent fiber residue	Section Determination of Conventional Nutrients; Van Soest et al. [20]
Starch	Perchloric acid extraction followed by anthrone colorimetric determination	Section Determination and Calculation of CNCPS Fractions
Non-protein nitrogen	Selective precipitation of true protein, followed by determination of nitrogen remaining in the soluble fraction	Section Determination and Calculation of CNCPS Fractions; Van Soest et al. [20]
Soluble crude protein	Buffer extraction of the soluble protein fraction, followed by nitrogen determination	Section Determination and Calculation of CNCPS Fractions; Van Soest et al. [20]
Neutral detergent insoluble protein	Determination of nitrogen remaining in the neutral detergent fiber residue	Section Determination and Calculation of CNCPS Fractions; Van Soest et al. [20]
Acid detergent insoluble protein	Determination of nitrogen remaining in the acid detergent fiber residue	Section Determination and Calculation of CNCPS Fractions; Van Soest et al. [20]

**Table A7:** Amino acid composition of ruminal microbial cell-wall and non-cell-wall protein (g/100 g protein).

Amino Acid	Cell Wall	Non-Cell Wall	Ruminal Microbes	
			Mean	SD
Met	2.40	2.68	2.60	0.70
Lys	5.60	8.20	7.90	0.90
His	1.74	2.69	2.00	0.40
Phe	4.20	5.16	5.10	0.30
Trp	1.63	1.63	-	-
Thr	3.30	5.59	5.80	0.50
Leu	5.90	7.51	8.10	0.80
Ile	4.00	5.88	5.70	0.40
Val	4.70	6.16	6.20	0.60
Arg	3.82	6.96	5.10	0.70

**Table A8:** Exploratory regression relationships between conventional nutrient components and CNCPS fractions.

Response Variable	Regression Equation	R^2^	*p*-Value
PB2 (% CP)	PB2 = 19.423 + 1.449 CP (% DM)	0.408	<0.01
PC (% CP)	PC = 7.423 − 0.272 CP (% DM) + 0.811 Ash (% DM)	0.781	<0.01
CA (% CHO)	CA = 5.698 + 0.632 CHO (% DM)− 1.266 ADF (% DM)	0.675	<0.01
CB1 (% CHO)	CB1 = 13.436 + 0.743 CHO (% DM) − 0.693 NDF (% DM) − 0.588 ADF (% DM)	0.763	<0.01
CB2 (% CHO)	CB2 = −5.072 + 1.615 NDF (% DM) − 0.993 ADF (% DM)	0.754	<0.01
CC (% CHO)	CC = −10.891 + 0.505 ADF (% DM) + 2.532 EE (% DM)	0.762	<0.01

*n* = 19. R^2^, coefficient of determination. These equations represent exploratory in-sample relationships and were not externally validated.

## References

[1] Higgs R.J., Chase L.E., Schwab C.G., Sloan B., Luchini D., LaPierre P.A., Van Amburgh M.E. (2023). Balancing dairy cattle diets for rumen nitrogen and methionine or all essential amino acids relative to metabolizable energy. J. Dairy Sci..

[2] Lee C., Hristov A.N., Heyler K.S., Cassidy T.W., Lapierre H., Varga G.A., Parys C. (2012). Effects of metabolizable protein supply and amino acid supplementation on nitrogen utilization, milk production, and ammonia emissions from manure in dairy cows. J. Dairy Sci..

[3] Menezes G.L., Letelier P., Dorea J.R.R., Sullivan M.L., Reinhardt L.A., Zanton G.I., Wattiaux M.A. (2026). Cow-level evaluation of plasma essential amino acid clusters and their association with lactating dairy cow performance and efficiency. J. Dairy Sci..

[4] Hristov A.N., Bannink A., Crompton L.A., Huhtanen P., Kreuzer M., McGee M., Nozière P., Reynolds C.K., Bayat A.R., Yáñez-Ruiz D.R. (2019). Invited review: Nitrogen in ruminant nutrition: A review of measurement techniques. J. Dairy Sci..

[5] Park K., Kim J., Hu H., Kambara M., Lee C., Hristov A.N. (2026). Effects of reduced metabolizable protein diets supplemented with rumen-protected His under adequate Met and Lys supply on production, nutrient digestibility, N utilization, and manure ammonia emissions in lactating cows. J. Dairy Sci..

[6] Sniffen C.J., O’Connor J.D., Van Soest P.J., Fox D.G., Russell J.B. (1992). A net carbohydrate and protein system for evaluating cattle diets: II. Carbohydrate and protein availability. J. Anim. Sci..

[7] Higgs R.J., Chase L.E., Ross D.A., Van Amburgh M.E. (2015). Updating the Cornell Net Carbohydrate and Protein System feed library and analyzing model sensitivity to feed inputs. J. Dairy Sci..

[8] Binggeli S., Lapierre H., Lemosquet S., Ouellet D.R., Pellerin D. (2022). Comparison of feed evaluation models on predictions of milk protein yield on Québec commercial dairy farms. J. Dairy Sci..

[9] Benchaar C., Hassanat F., Beauchemin K.A., Ouellet D.R., Lapierre H., Côrtes C. (2023). Effect of metabolizable protein supply on milk performance, ruminal fermentation, apparent total-tract digestibility, energy and nitrogen utilization, and enteric methane production of ayrshire and holstein cows. Animals.

[10] Anger J.-C., Loncke C., Bidaux R., Dieho K., Nozière P., Lemosquet S., van Milgen J. (2026). Lactation persistency of dairy cows fed low metabolizable protein diets with two different starch contents and degradability levels crossed with rumen-protected amino acid supplies. J. Dairy Sci..

[11] Coblentz W.K., Fritz J.O., Fick W.H. (1998). In situ dry matter, nitrogen, and fiber degradation of alfalfa, red clover, and eastern gamagrass at four maturities. J. Dairy Sci..

[12] Morales Á.G., Navarro Á.R., Pulido R.G., Hanigan M.D. (2024). Characterization of in situ ruminal degradation of crude protein and individual amino acids from ryegrass. Agriculture.

[13] Taghavi M., Taghizadeh A., Mehmannavaz Y., Hoseinkhani A., Mohammadzadeh H., Macit M., Palangi V., Lackner M. (2023). Degradability of Vicia ervilia Grain Using in Situ and CNCPS Methods, and Model-Based Analysis of Its Ruminal Degradation. Fermentation.

[14] (2014). Determination of Moisture in Feedstuffs.

[15] (2018). Determination of Crude Protein in Feed—Kjeldahl Method.

[16] (2022). Determination of Neutral Detergent Fibre in Feeds.

[17] (2022). Determination of Acid Detergent Fiber (ADF) in Feeds.

[18] (2007). Determination of Ash in Feeds.

[19] (2006). Determination of Crude Fat in Feeds.

[20] Van Soest P.J., Robertson J.B., Lewis B.A. (1991). Methods for dietary fiber, neutral detergent fiber, and nonstarch polysaccharides in relation to animal nutrition. J. Dairy Sci..

[21] Beckers Y., Théwis A., Maudoux B., François E. (1995). Studies on the in situ nitrogen degradability corrected for bacterial contamination of concentrate feeds in steers. J. Anim. Sci..

[22] Legay-Carmier F., Bauchart D. (1989). Distribution of bacteria in the rumen contents of dairy cows given a diet supplemented with soya-bean oil. Br. J. Nutr..

[23] Krawielitzki K., Schmidt T., Voigt J., Kowalczyk J., Gabel M. (2006). Dynamics of microbial contamination of protein during ruminal in situ incubation of feedstuffs. J. Anim. Feed Sci..

[24] Ørskov E.R., McDonald I. (1979). The estimation of protein degradability in the rumen from incubation measurements weighted according to rate of passage. J. Agric. Sci..

[25] NRC (2001). Nutrient Requirements of Dairy Cattle: 2001.

[26] Seo S., Tedeschi L.O., Schwab C.G., Garthwaite B.D., Fox D.G. (2006). Evaluation of the passage rate equations in the 2001 Dairy NRC model. J. Dairy Sci..

[27] NASEM (2021). Nutrient Requirements of Dairy Cattle: 2021.

[28] Clark J.H., Klusmeyer T.H., Cameron M.R. (1992). Symposium: Nitrogen metabolism and amino acid nutrition in dairy cattle. Microbial protein synthesis and flows of nitrogen fractions to the duodenum of dairy cows. J. Dairy Sci..

[29] O’Connor J.D., Sniffen C.J., Fox D.J. (1993). A net carbohydrate and protein system for evaluating cattle diets: IV. Predicting amino acid adequacy. J. Anim. Sci..

[30] Schwab C.G., Tylutki T.P., Ordway R.S., Sheaffer C., Stern M.D. (2003). Characterization of Proteins in Feeds. J. Dairy Sci..

[31] Yang T., Zou X., Zhai D., Wang X., Guo Z., Hou Q., Zhao W., Zhao M. (2025). Effect of electro-stimulated Bacillus subtilis and Lactobacillus casei on ensiling quality, anti-nutrients, and bacterial community of mulberry leaf silage. J. Sci. Food Agric..

[32] Zhang X., Ke W.C., Ding Z.T., Xu D.M., Wang M.S., Chen M.Y., Guo X.S. (2022). Microbial mechanisms of using feruloyl esterase-producing Lactobacillus plantarum A1 and grape pomace to improve fermentation quality and mitigate ruminal methane emission of ensiled alfalfa for cleaner animal production. J. Environ. Manag.

[33] Culman S., Pinto P., Pugliese J., Crews T., DeHaan L., Jungers J., Larsen J., Ryan M., Schipanski M., Sulc M. (2023). Forage harvest management impacts “Kernza” intermediate wheatgrass productivity across North America. Agron. J..

[34] Lang X., Yang M., Saleem A.M., Zhao X., Xu H., Li Y., Xu R., Cao J., Xu C., Cui Y. (2022). Nutritional Value, Fermentation Characteristics and In Vitro Degradability of Whole Wheat Hay Harvested at Three Stages of Maturity. Animals.

[35] Głazowska S., Gundersen E., Heiske S., Lübeck M., Mravec J., Jørgensen B. (2023). Tracking digestible and non-digestible cell wall components during protein concentrate production from grass-clover and alfalfa. Biomass Convers. Biorefin..

[36] Eldeeb M.A., Hohman G., Shahid M. (2025). Novel Approaches in Targeting Cell Surface and Secreted Proteins for Lysosomal Degradation. ChemBioChem.

[37] Van Soest P.J. (1994). Nutritional Ecology of the Ruminant.

[38] Bachmann M., Kuhnitzsch C., Okon P., Martens S.D., Greef J.M., Steinhöfel O., Zeyner A. (2019). Ruminal In Vitro Protein Degradation and Apparent Digestibility of Energy and Nutrients in Sheep Fed Native or Ensiled + Toasted Pea (*Pisum sativum*) Grains. Animals.

[39] Chen L., Bao X., Guo G., Huo W., Xu Q., Wang C., Li Q., Liu Q. (2021). Effects of Hydrolysable Tannin with or without Condensed Tannin on Alfalfa Silage Fermentation Characteristics and In Vitro Ruminal Methane Production, Fermentation Patterns, and Microbiota. Animals.

[40] Chalupa W., Sniffen C.J. (1996). Protein and amino acid nutrition of lactating dairy cattle—Today and tomorrow. Anim. Feed Sci. Technol..

[41] Silva S.P.D., Rodrigues M.T., Vieira R.A.M., Silva M.M.C.D. (2013). In vitro degradation kinetics of protein and carbohydrate fractions of selected tropical forages = Cinética de degradao in vitro das fraes de proteína e carboidrato em forrageiras tropicais. Biosci. J..

[42] Cui Y.C. (2012). Study on the Digestibility of Protein Fraction C of Dairy Cows Feedstuffs. Master’s Dissertation.

[43] Zhang C., Tu Y., Ma T., Diao Q. (2022). Connection between Circadian Rhythm and Rumen Digestibility of Concentrate and Roughage in Sheep. Agriculture.

[44] Nocek J.E., Tamminga S. (1991). Site of digestion of starch in the gastrointestinal tract of dairy cows and its effect on milk yield and composition. J. Dairy Sci..

[45] Herrera-Saldana R.E., Huber J.T., Poore M.H. (1990). Dry matter, crude protein, and starch degradability of five cereal grains. J. Dairy Sci..

[46] Gu J.X. (2005). The Rumen Degradation and Utilization of Corn Starch and Growth of Hu-Sheep Fed on it as Influenced by Corn Processing. Master’s Dissertation.

[47] Retnani Y., Risyahadi S.T., Qomariyah N., Barkah N.N., Taryati T., Jayanegara A. (2022). Comparison between pelleted and unpelleted feed forms on the performance and digestion of small ruminants: A meta-analysis. J. Anim. Feed Sci..

[48] Jiang F. (2009). Evaluation of Nutritive Value of Commom Feedstuffs for Ruminants and Variation Analysis. Master’s Dissertation.

[49] Russell J.B., Sniffen C.J., Van Soest P.J. (1983). Effect of carbohydrate limitation on degradation and utilization of casein by mixed rumen bacteria. J. Dairy Sci..

[50] Edmunds B., Südekum K.-H., Bennett R., Schröder A., Spiekers H., Schwarz F.J. (2013). The amino acid composition of rumen-undegradable protein: A comparison between forages. J. Dairy Sci..

[51] Zhang Y.X. (2012). Study on Predicting Model of the Absorbed Amino Acid Fluxes in the Small Intestine of Milking Cow. Master’s Dissertation.

[52] Pacheco D., Patton R.A., Parys C. (2012). Ability of commercially available dairy ration programs to predict duodenal flows of protein and essential amino acids in dairy cows. J. Dairy Sci..

[53] Lanzas C., Tedeschi L.O., Seo S. (2007). Evaluation of protein fractionation systems used in formulating rations for dairy cattle. J. Dairy Sci..

[54] Martineau R., Ouellet D.R., Pellerin D., Firkins J.L., Hanigan M.D., White R.R., LaPierre P.A., Van Amburgh M.E., Lapierre H. (2024). Ability of three dairy feed evaluation systems to predict postruminal outflows of amino acids in dairy cows: A meta-analysis. J. Dairy Sci..

[55] Martineau R., Ouellet D.R., Pellerin D., LaPierre P.A., Van Amburgh M.E., Lobley G.E., Lapierre H. (2025). Net portal appearance used to assess feed evaluation system predictions of the digestive flow and gut metabolism of essential amino acids in dairy cows: A meta-analysis. J. Dairy Sci..

[56] Van Duinkerken G., Blok M.C., Bannink A., Cone J.W., Dijkstra J., Van Vuuren A.M., Tamminga S. (2011). Update of the dutch protein evaluation system for ruminants: The DVE/OEB2010 system. J. Agric. Sci..

[57] Killerby M.A., de Souza G.M., Ruh-Etteldorf K.E., Pszczolkowski V., Hoppmann A., Cohan E., Danes M.A.C., Arriola Apelo S.I. (2026). Insulinemic energy and amino acids supplied postruminally independently regulate the performance and metabolism of peak-lactation dairy cows. J. Dairy Sci..

[58] Hume I.D., Moir R.J., Somers M. (1970). Synthesis of microbial protein in the rumen. I. Influence of the level of nitrogen intake. Aust. J. Agric. Res..

[59] Olson K.C., Cochran R.C., Jones T.J., Vanzant E.S., Titgemeyer E.C., Johnson D.E. (1999). Effects of ruminal administration of supplemental degradable intake protein and starch on utilization of low-quality warm-season grass hay by beef steers. J. Anim. Sci..

