# Supplementary material for: CNCPS-Based Nutrient Fractionation, Ruminal Degradation Kinetics, and Intestinal Amino Acid Flow Estimates in Dairy Feedstuffs
Source: Animals (Basel). 2026 Aug 3;16(15):2371. doi: 10.3390/ani16152371 (PMC13465355; doi:10.3390/ani16152371)
Supplement: Supplementary file 1 [file animals-16-02371-s001.zip › animals-4444963-supplementary.pdf]

Table S1. Excluded exploratory regression models for CNCPS protein fractions.

Response variable	Regression equation	R^2	p-value	Reason for exclusion
PA	PA (% CP) = −417.435 + 4.891 DM (% as-fed)	0.2857	<0.05	Low explanatory power and limited biological interpretability of DM as the sole predictor
PB1	PB1 (% CP) = 141.020 − 1.463 DM (% as-fed)	0.2419	<0.05	Low explanatory power and limited biological interpretability of DM as the sole predictor
PB3	PB3 (% CP) = 168.195 − 1.835 DM (% as-fed) − 0.108 CP (% DM) + 0.426 NDF (% DM) − 0.332 ADF (% DM)	0.2341	>0.05	Low explanatory power and nonsignificant overall model

Note: n = 19. DM is expressed as % as-fed; CP, NDF, and ADF are expressed as % DM; PA, PB1, and PB3 are expressed as % CP. R^2, coefficient of determination. These models were excluded from the main results because of low explanatory power, limited biological interpretability, or lack of statistical significance.

Table S2. Complete measured amino acid composition of insoluble protein in the tested feed samples (g/100 g insoluble protein).

**Item**	**Soybean meal**	**Wheat**	**DDGS**	**Molassed soybean hulls**	**Extruded Soybean hulls**	**Wheat middlings**	**Alfalfa**
Lys	2.911	0.792	0.719	0.649	0.533	0.486	0.774
Met	0.261	0.333	0.366	0.218	0.249	0.242	0.25
Arg	3.068	0.416	0.986	0.408	0.239	0.735	0.569
His	1.348	0.372	0.717	0.249	0.181	0.346	0.281
Ile	2.216	0.446	0.952	0.349	0.230	0.405	0.597
Leu	3.727	0.673	3.003	0.555	0.354	0.751	0.955
Phe	4.093	1.632	3.736	1.279	1.298	1.373	1.537
Thr	1.817	0.453	0.896	0.337	0.203	0.38	0.552
Trp	0.940	0.130	0.230	0.230	0.770	0.210	0.210
Val	3.952	1.818	3.106	1.521	1.443	1.717	2.061
EAA	24.33	7.065	14.71	5.795	5.500	6.645	7.786
Asp	5.204	0.860	1.702	0.846	0.570	0.755	1.234
Ser	2.027	0.425	0.939	0.456	0.315	0.452	0.452
Glu	8.608	1.664	4.330	1.396	0.658	2.732	1.329
Gly	1.649	0.487	0.807	0.668	0.636	0.593	0.658
Ala	1.524	0.489	1.510	0.389	0.285	0.542	0.665
Cys	0.234	0.213	0.210	0.218	0.177	0.220	0.234
Tyr	0.788	-	0.375	-	-	-	0.110
Pro	2.757	0.911	2.349	0.794	0.685	1.188	0.87
NEAA	22.79	5.049	12.22	4.767	3.326	6.482	5.552
TAA	47.12	12.11	26.93	10.56	8.826	13.13	13.34

Table S2. (continued)

**Item**	**Wheat bran**	**Flaked corn**	**Cottonseed 1**	**Cottonseed 2**	**Pelleted sugar beet pulp**	**Whole-plant corn silage 1**
Lys	0.505	0.166	0.702	0.701	0.700	0.069
Met	0.267	0.247	0.344	0.256	0.277	0.24
Arg	0.873	0.208	1.687	1.589	0.345	0.047
His	0.42	0.185	0.485	0.446	0.281	0.048

Ile	0.424	0.188	0.501	0.535	0.377	0.083
Leu	0.787	0.736	0.850	0.925	0.543	0.193
Phe	1.397	1.335	1.971	2.950	1.262	1.077
Thr	0.34	0.204	0.508	0.520	0.418	0.066
Trp	0.160	0.120	0.250	0.250	0.110	0.110
Val	1.81	1.517	2.717	2.535	1.734	1.154
EAA	6.983	4.906	10.02	10.71	6.047	3.087
Asp	0.829	0.377	1.481	1.487	0.78	0.155
Ser	0.405	0.246	0.549	0.555	0.364	0.056
Glu	2.592	1.235	3.394	3.323	1.081	0.205
Gly	0.707	0.211	0.656	0.67	0.402	0.101
Ala	0.612	0.396	0.629	0.67	0.434	0.111
Cys	0.208	0.201	0.224	0.226	0.209	0.120
Tyr	-	-	0.145	0.097	0.075	-
Pro	1.305	0.843	1.068	0.977	0.693	-
NEAA	6.658	3.509	8.146	8.005	4.038	0.748
TAA	13.64	8.415	18.16	18.71	10.09	3.835

Table S2. (continued)

Item	Whole-plant corn silage 2	Corn stover silage	Wheat straw	Dried brewer grains	Dried distiller grains 1	Dried distiller grains 2
Lys	0.078	0.109	0.051	0.239	0.782	0.648
Met	0.233	0.243	0.297	0.279	0.32	0.264
Arg	0.065	0.072	-	0.366	1.056	0.781
His	0.054	0.051	-	0.387	0.485	0.402
Ile	0.095	0.111	0.032	0.285	0.848	0.757
Leu	0.225	0.21	0.093	0.688	1.513	1.357
Phe	1.066	1.149	1.342	1.225	1.742	3.008
Thr	0.081	0.087	0.039	0.343	0.681	0.586
Trp	0.120	0.100	0.100	0.110	0.270	0.480
Val	1.142	1.256	1.54	1.872	2.489	2.794
EAA	3.159	3.388	3.494	5.794	10.186	11.077
Asp	0.183	0.214	0.116	0.487	1.502	1.238
Ser	0.068	0.065	0.039	0.289	0.677	0.559
Glu	0.28	0.225	0.113	1.618	4.033	3.721
Gly	0.119	0.128	0.053	0.53	0.797	0.669
Ala	0.127	0.13	0.044	0.476	0.987	0.811
Cys	0.144	0.171	0.110	0.110	0.244	-
Tyr	-	-	-	0.09	0.191	0.130
Pro	-	-	-	1.401	2.129	2.149
NEAA	0.921	0.933	0.475	5.001	10.56	9.277
TAA	4.08	4.321	3.969	10.80	20.75	20.35

"-" indicates that the amino acid was not detected under the analytical conditions used; it does not represent a missing value or a confirmed zero concentration. For calculation of EAA, NEAA, and TAA totals, nondetected values were treated as zero.
